# Magneto-structural correlations in arsenic- and selenium-ligated dysprosium single-molecule magnets†
†Electronic supplementary information (ESI) available: Synthetic details, spectroscopic characterization for all compounds, X-ray crystallography details and crystallographic information files, computational details. CCDC 1403610–1403614. For ESI and crystallographic data in CIF or other electronic format see DOI: 10.1039/c5sc03755g


**DOI:** 10.1039/c5sc03755g

**Published:** 2015-12-15

**Authors:** Thomas Pugh, Veacheslav Vieru, Liviu F. Chibotaru, Richard A. Layfield

**Affiliations:** a School of Chemistry , The University of Manchester , Oxford Road , Manchester , M13 9PL , UK . Email: Richard.Layfield@manchester.ac.uk; b Theory of Nanomaterials Group , Katholieke Universiteit Leuven , Celestijenlaan 200F , 3001 Heverlee , Belgium

## Abstract

The structures and magnetic properties of the arsenic- and selenium-ligated dysprosium single-molecule magnets (SMMs) [Cp′_3_Dy(AsH_2_Mes)] (**3-Dy**), [(η^5^-Cp′_2_Dy){μ-As(H)Mes}]_3_ (**4-Dy**), [Li(thf)_4_]_2_[(η^5^-Cp′_2_Dy)_3_(μ_3_-AsMes)_3_Li] ([Li(thf)_4_]_2_[**5-Dy**]), and [(η^5^-Cp′_2_Dy){μ-SeMes}]_3_ (**6-Dy**) are described. The arsenic-ligated complexes **4-Dy** and **5-Dy** are the first SMMs to feature ligands with metalloid elements as the donor atoms. The arsenide-ligated complex **4-Dy** and the selenolate-ligated complex **6-Dy** show large anisotropy barriers in the region of 250 cm^–1^ in zero d.c. field, increasing to 300 cm^–1^ upon 5% magnetic dilution. Theoretical studies reveal that thermal relaxation in these SMMs occurs *via* the second-excited Kramers' doublet. In contrast, the arsinidene-ligated SMM **5-Dy** gives a much smaller barrier of 23 cm^–1^, increasing to 35 cm^–1^ upon dilution. The field-dependence of the magnetization for **4-Dy** and **5-Dy** at 1.8 K show unusual plateaus around 10 kOe, which is due to the dominance of arsenic-mediated exchange over the dipolar exchange. The effects of the exchange interactions are more pronounced in **5-Dy**, which is a consequence of a small but significant increase in the covalent contribution to the predominantly ionic dysprosium-arsenic bonds. Whereas the magnetically non-dilute dysprosium SMMs show only very narrow magnetization *versus* field hysteresis loops at 1.8 K, the impact of magnetic dilution is dramatic, with butterfly-shaped loops being observed up to 5.4 K in the case of **4-Dy**. Our findings suggest that ligands with heavier p-block element donor atoms have considerable potential to be developed more widely for applications in molecular magnetism.

## Abstract 2

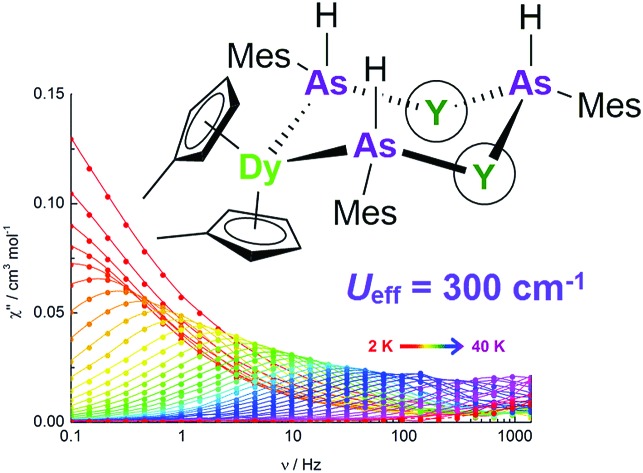
A series of arsine-, arsenide, arsinidene and selenium-ligated dysprosium single-molecule magnets (SMMs) are described. The arsenide- and selenolate-ligated SMMs show anisotropy barriers up to 300 cm^–1^ upon magnetic dilution.

## Introduction

The strong magnetic anisotropy of certain lanthanide and transition metal ions has enabled the development of a vast array of single-molecule magnets (SMMs). SMMs are a type of molecular nanomagnet characterized by the ability to display magnetic hysteresis that is molecular in origin, and by an effective energy barrier (*U*_eff_) to reversal of the magnetization.^1^–^5^ In addition to the fundamental interest in the electronic structure of SMMs, the electron-transport properties of these materials have also stimulated considerable interest by virtue of their potential applications in molecular spintronics.^6^,^7^ Despite the remarkable progress that has been made with SMMs, a number of challenges remain. Firstly, SMMs only function when cooled with cryogenic liquids, hence there is a need to increase the temperatures at which they function. Secondly, investigating a wider range of SMMs at the single-molecule level, on surfaces or in junctions, will be important for developing the field.^8^,^9^

The fact that SMMs typically function at very low temperatures is due to the availability of myriad mechanisms through which reversal of the magnetization may occur.^10^ Ultimately, therefore, there is a need to understand why certain relaxation processes in SMMs are so facile, and hence to use innovative coordination chemistry to address these processes. To date, successful strategies have included: studying metal ions in high-symmetry coordination environments;^11^–^15^ the use of radical bridging ligands to suppress quantum tunnelling of the magnetization (QTM);^16^ the use of magnetic dilution to eliminate dipolar exchange interactions;^17^,^18^ modification of the crystal field through changes to the inductive effects of the ligand substituents;^19^ the assembly of single-ion magnet building blocks into extended SMMs;^20^ the synthesis of metal–organic frameworks in which SMMs are used as nodes^21^ or loaded into the porous structure;^22^,^23^ and the use of 3d–4f exchange interactions to enhance the blocking temperature.^24^

The overwhelming majority of SMMs contain ligands based on 2p elements, with N- and O-donor ligands being particularly prevalent,^1^–^5^ but with organometallic ligands now growing in popularity.^25^–^30^ An alternative strategy with hugely under-exploited potential for enhancing the properties of SMMs is to use ligands with heavier p-block elements as the donor atoms. More generally, molecular magnets containing, for example, heavier pnictogen (P–Bi) donor ligands are extremely rare.^31^ Elements such as arsenic and selenium offer more diffuse valence orbitals than their lighter congeners, which introduces possibilities for influencing the magnetic properties of lanthanides through greater covalent contributions to the predominantly ionic metal–ligand bonds,^32^ which could in turn enable stronger magnetic exchange. To explore these ideas, we now describe the first arsenic- and selenium-ligated SMMs, *i.e.* the dysprosium-arsine [Cp′_3_Dy(AsH_2_Mes)], the dysprosium arsenide [(η^5^-Cp′_2_Dy){μ-As(H)Mes}]_3_, the dysprosium arsinidene [Li(thf)_4_]_2_[(η^5^-Cp′_2_Dy)_3_(μ_3_-AsMes)_3_Li] and the dysprosium selenolate [(η^5^-Cp′_2_Dy){μ-SeMes}]_3_·toluene (Cp′ = methylcyclopentadienyl).

## Results and discussion

**Scheme 1:**
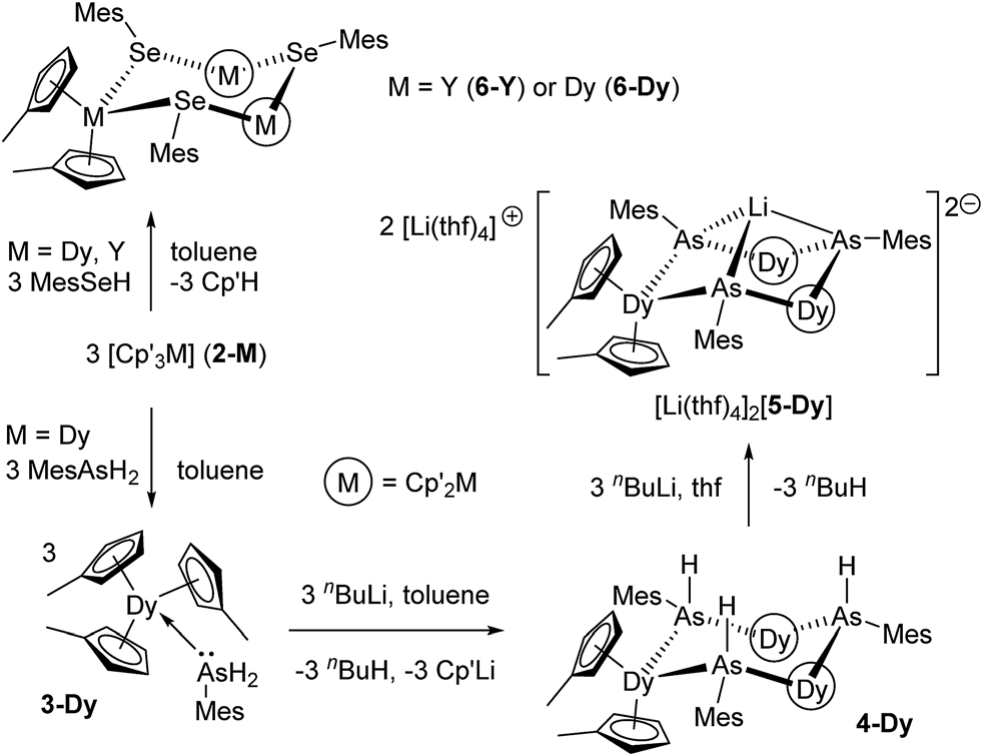
Synthesis of **3-Dy**, **4-Dy**, [Li(thf)_4_]_2_[**5-Dy**] and **6-M** (M = Y, Dy).

The addition of mesitylarsine (MesAsH_2_, **1**) to Cp′_3_Dy (**2-Dy**) produced [Cp′_3_Dy(AsH_2_Mes)] (**3-Dy**) in 83% yield (Scheme 1). The dysprosium arsenide [(η^5^-Cp′_2_Dy){μ-As(H)Mes}]_3_·toluene (**4-Dy**·toluene) was synthesized in a yield of 66% by deprotonation of **3-Dy** with one stoichiometric equivalent of ^*n*^BuLi in toluene. The dysprosium arsinidene [Li(thf)_4_]_2_[(η^5^-Cp′_2_Dy)_3_(μ_3_-AsMes)_3_Li]·thf, [Li(thf)_4_]_2_[**5-Dy**]·thf, was obtained by deprotonation of **4-Dy** with three equivalents of ^*n*^BuLi in thf, and was isolated in a yield of 77%. The dysprosium selenolate complex [(η^5^-Cp′_2_Dy){μ-SeMes}]_3_·toluene (**6-Dy**) was synthesized in yield of 91% by the deprotonation of mesitylselenol by Cp′_3_Dy in toluene. The analogous yttrium selenolate (**6-Y**), which was synthesized for the purpose of magnetic dilution experiments (see below), was obtained in an identical manner to the dysprosium version using Cp′_3_Y (**2-Y**) in 79% yield (Scheme 1).

**Fig. 1:**
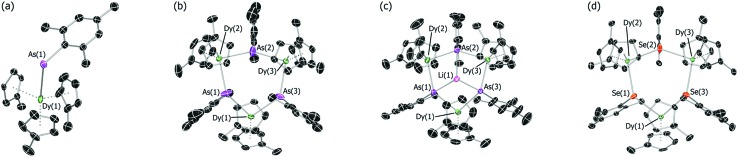
Thermal ellipsoid representations (50% probability) of the molecular structures of: (a) **3-Dy**; (b) **4-Dy**; (c) **5-Dy** and; (d) **6-Dy**. For clarity, hydrogen atoms are omitted.

The structures of all five compounds were determined by X-ray crystallography (Fig. 1, Tables 1 and S1†). In **3-Dy**, the centroids of the three η^5^-Cp′ ligands define an approximate trigonal plane, with the dysprosium-arsenic bond occupying a position approximately perpendicular to this plane. The Dy–C and Dy–As distances in **3-Dy** are 2.665(5)–2.759(15) Å (average 2.686 Å) and 3.0869(6) Å, respectively. The structure of **4-Dy** consists of a central chair-like Dy_3_As_3_ ring with Dy–As distances of 2.9840(18)–3.0088(18) Å (average 2.997 Å), and with As–Dy–As and Dy–As–Dy angles in the range 91.82(2)–95.13(2)° and 131.97(2)–136.24(2)°, respectively. Each dysprosium centre in **4-Dy** is complexed by two η^5^-Cp′ ligands, producing Dy–C distances of 2.597(15)–2.689(13) Å (average 2.636 Å). The structure of the dianion **5-Dy** is similar to that of **4-Dy** but features much shorter (by 0.128 Å, on average) Dy–As distances of 2.8515(6)–2.8908(7) Å (average 2.869 Å). The Dy–C distances in **5-Dy** are 2.633(6)–2.747(3) Å (average 2.680 Å), and the As–Li distances are 2.541(6)–2.627(6) Å.

The dysprosium selenolate complex **6-Dy** also features a Dy_3_Se_3_ ring in a chair conformation, with Dy–Se distances in the range 2.9083(15)–2.9330(17) Å (average 2.918 Å), hence they are shorter than the Dy–As distances in **4-Dy** by 0.079 Å. The Se–Dy–Se and Dy–Se–Dy angles are 95.53(2)–100.17(2)° and 130.37(2)–133.55(2)°, respectively. The two η^5^-Cp′ ligands per dysprosium in **6-Dy** produce Dy–C distances of 2.599(12)–2.688(13) Å (average 2.632 Å), which is the same range as the analogous distances in **4-Dy**. The molecular structure of **6-Y** is essentially the same as that of the dysprosium analogue (Table 1, Fig. S4†). The diamagnetism of **6-Y** also allowed the structure to be characterized in toluene-d_8_ solution by ^1^H NMR spectroscopy (Fig. S1†), where it was found to be consistent with the solid-state structure.

**Table 1:** Selected bond lengths [Å] and angles [°] for **3-Dy**, **4-Dy**, **5-Dy**, **6-Dy** and **6-Y**

	**3-Dy**	**4-Dy**	**5-Dy**	**6-Dy**	**6-Y**
M–E	3.0869(6)	2.9840(18)–3.0088(18)	2.8515(6)–2.8908(7)	2.9083(15)–2.9330(17)	2.8992(6)–2.9246(7)
M–C	2.665(5)–2.759(15)	2.597(15)–2.689(13)	2.633(6)–2.747(3)	2.599(12)–2.688(13)	2.593(4)–2.672(4)
M–Cp_cent_	2.415(2)–2.441(2)	2.340(7)–2.364(6)	2.373(2)–2.426(2)	2.336(5)–2.355(6)	2.325(2)–2.355(2)
Li–As			2.541(6)–2.627(6)		
M···M		5.4433(10)–5.5362(8)	5.2574(8)–5.3011(6)	5.2911(11)–5.3675(9)	5.2968(6)–5.3503(5)
E–M–E		89.46(7)–96.77(6)	91.82(2)–95.13(2)	95.43(5)–100.95(5)	95.53(2)–100.17(2)
M–E–M		129.71(6)–135.04(7)	131.97(2)–136.24(2)	129.86(5)–133.65(6)	130.37(2)–133.55(2)
As–Li–As			107.3(2)–110.1(2)		
Dy–As–Li			76.18(14)–80.01(15)		

Rare-earth complexes of arsenic donor ligands are uncommon.^33^ The most closely related compounds to **4-Dy** are the samarium arsenide complexes [Cp*_2_Sm(AsPh_2_)(L)] (L = thf or nothing), which were formed by samarium(ii) reduction of Ph_2_As–AsPh_2_.^34^ Complex **5-Dy** is only the second rare-earth complex of an arsinidene (RAs^2–^) ligand, with the first example being the yttrium complex anion [(η^5^-Cp′_2_Y)_3_(μ_3_-AsMes)_3_Li]^2–^ (**5-Y**),^35^ however **5-Dy** is the first lanthanide (4f) complex of such a ligand. Selenolate-bridged lanthanide complexes of the type [Cp_2_Ln(μ-SeR)]_*n*_ are more numerous,^36^ however their magnetic properties have not been described.

### Static-field (d.c.) magnetic properties

The static-field (d.c.) magnetic susceptibilities of **3-Dy**, **4-Dy**·toluene, [Li(thf)_4_]_2_[**5-Dy**]·thf and **6-Dy**·toluene were measured in the temperature range 2–300 K on polycrystalline samples restrained in eicosane. An applied field of *H*_dc_ = 1 kOe was used for each measurement. All four compounds behave as expected, with *χ*_M_*T* values at 300 K of 13.31, 43.55, 41.78 and 42.91 cm^3^ K mol^–1^, respectively, which are close to the predicted values of 14.17 cm^3^ K mol^–1^ for a single dysprosium ion and 42.51 cm^3^ K mol^–1^ for three uncorrelated dysprosium ions, each with a ^6^H_15/2_ ground term and *g* = 4/3 (Fig. S5†).^37^ The *χ*_M_*T* values decrease gradually in each case as the temperature is lowered, reaching 7.64, 18.07, 14.81 and 16.54 cm^3^ K mol^–1^, respectively, at 2 K. In the case of **3-Dy**, the steep decrease below about 25 K is due to depopulation of the excited *m*_*J*_ sub-levels arising from the crystal field splitting, and in the three trimetallic complexes the decrease is likely due to a combination of crystal field effects and antiferromagnetic exchange. The much smaller low-temperature value of *χ*_M_*T* for **5-Dy** may indicate stronger exchange in the arsinidene-ligated complex (see below).

**Fig. 2:**
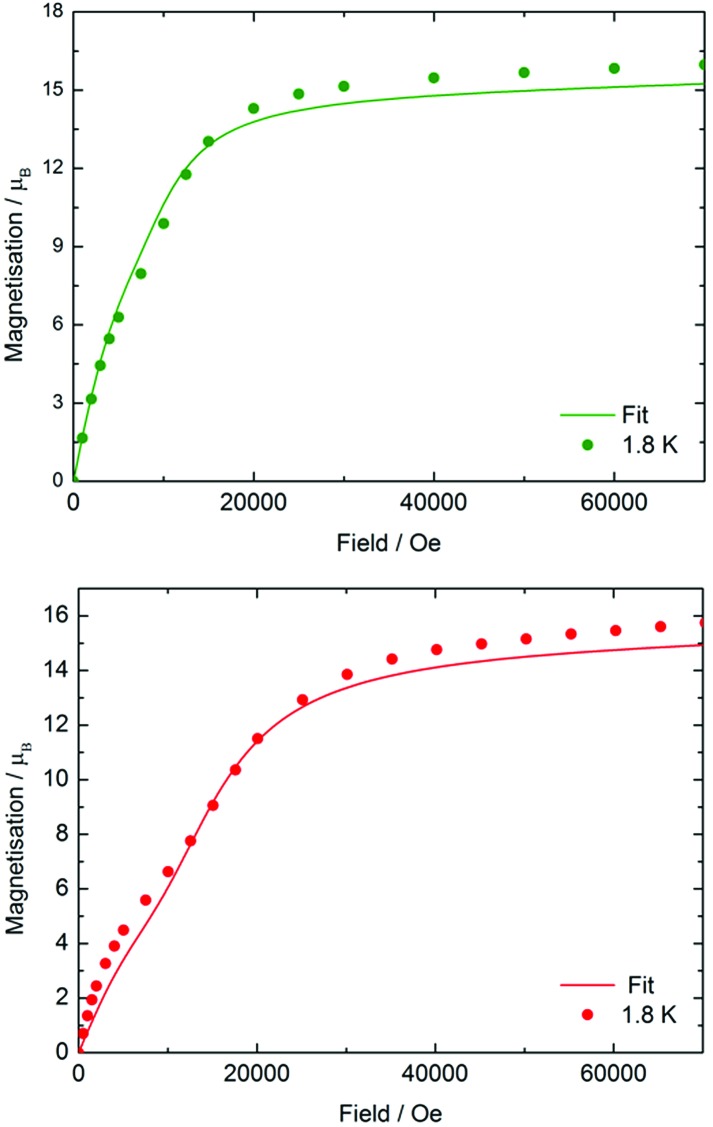
Experimental (circles) and calculated (solid lines) field dependence of the magnetization for **4-Dy** (upper) and **5-Dy** (lower) at 1.8 K.

The field dependence of the magnetization (*M*) was also measured for each compound at 1.8 K and 3.0 K using fields in the range *H* = 0–70 kOe (0–7 T) (Fig. S6†). The following discussion refers to the data collected at 1.8 K. In **3-Dy**, *M*(*H*) shows no unusual features, with the magnetization increasing rapidly as the field increases to 18 kOe. At higher fields, the magnetization increases more slowly to reach a value of 5.43 *μ*_B_ at 70 kOe, which is close to the expected value of *M* = 5.25 *μ*_B_ for a single dysprosium ion. The magnetization behaviour of the two arsenic-ligated trimetallic species is much more interesting (Fig. 2). The magnetization of **4-Dy** increases in a sharp, uniform manner as a field of 7.5 kOe is reached, and then a less uniform increase in magnetization is observed at about 10 kOe. The effect was seen more clearly at 1.8 K than at 3.0 K. At higher fields, the magnetization increases rapidly again, and above a field of approximately 15 kOe the increase in magnetization is slower and the saturation value of 15.97 *μ*_B_ is reached at 70 kOe, which is close to the expected value of 15.75 *μ*_B_ for three Dy^3+^ ions. The same general trend is observed in the *M*(*H*) data for **5-Dy**, however the non-uniform increase in the magnetization at lower fields is more pronounced. The magnetization in **5-Dy** also increases at a slower rate than that observed for **4-Dy**. For example, the magnetization of **4-Dy** at 1.8 K in a 10 kOe field is 9.88 *μ*_B_, rising to 14.30 *μ*_B_ in a 20 kOe field. In contrast, the corresponding magnetization values for **5-Dy** are 7.06 *μ*_B_ and 12.10 *μ*_B_. The gradient of the *M*(*H*) curve for **5-Dy** decreases markedly at fields in the range of *H* = 8–10 kOe before increasing again up to about 18 kOe. At higher values of the magnetic field the magnetization in **5-Dy** begins to saturate as in **4-Dy**, reaching a value of 15.76 *μ*_B_ at 70 kOe.

The features of the *M*(*H*) data for **4-Dy** and **5-Dy** suggest an antiferromagnetic ground state in relatively weak magnetic fields, which eventually gives way to a ferromagnetic ground state in stronger fields. The more pronounced effect in **5-Dy** can be rationalized on the grounds that the arsinidene ligand [MesAs]^2–^ enables stronger exchange interactions than the arsenide ligand [MesAs(H)]^–^. To provide support for this explanation, a theoretical study of the magnetic susceptibility and the magnetization was undertaken using *ab initio* calculations of the CASSCF/RASSI/SINGLE_ANISO type.^38^ In the calculations, the total magnetic interactions in the Dy_3_ compounds were accounted for by the following exchange Hamiltonian:
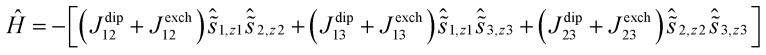


The total magnetic coupling between the three pairs of dysprosium centres in **4-Dy** and **5-Dy** can be expressed as a sum of the dipolar contribution and the exchange contribution, *i.e. J*_tot_ = *J*_dip_ + *J*_ex_. The values of *J*_dip_ were calculated exactly, and *J*_ex_ was obtained by fitting the experimental data. As fitting of magnetic susceptibility data with three exchange parameters is challenging, we initially ran broken-symmetry DFT calculations to estimate the values (Table S11†). By slightly varying the exchange parameters determined by DFT, we found the best sets of parameters that provide a good match to the experimental data (Fig. S29, S31 and S33†). The calculations reproduced the experimental magnetic susceptibility data accurately (Fig. S27, S29, S31 and S33†), with the discrepancy between experiment and theory being no greater than 4%. The experimental magnetization *vs.* field data were also reproduced reasonably well by the calculations and, crucially, in the case of **4-Dy** and **5-Dy**, the non-uniform increases in the magnetization in relatively small magnetic fields were also reproduced (Fig. 2, S28, S30, S32 and S34†).

**Fig. 3:**
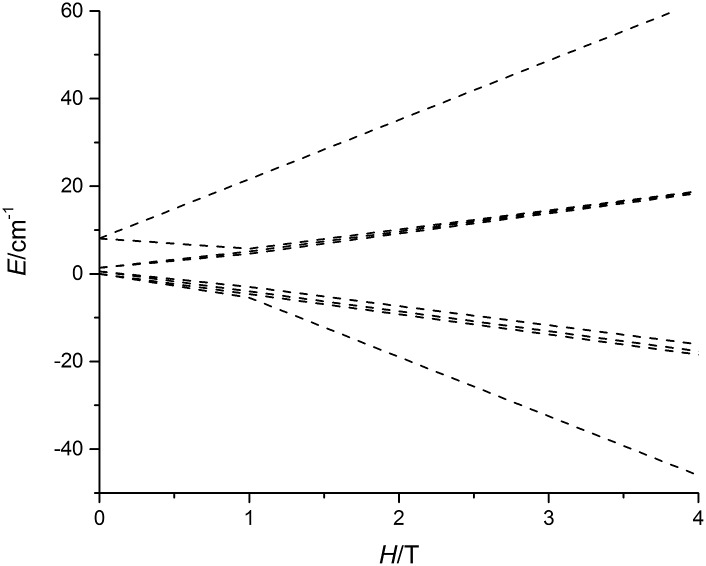
Calculated evolution of the lowest magnetic states of **5-Dy** with applied field. The field is applied along the main anisotropy axis of one of the Dy^3+^ ions.

The non-uniform increase in the magnetization is related to the intersection of the Zeeman levels arising from different exchange states. Fig. 3 shows that the Zeeman component of the second excited exchange level becomes the ground state at a field of approximately 1 Tesla applied perpendicular to the Dy_3_ plane in **5-Dy**, consistent with the experimental data. For the inflection to be observable, the exchange splitting should be sufficiently large, which is indeed the case for **5-Dy** according to the calculation (Table 2). The calculations show that the magnetic coupling in **4-Dy** and **5-Dy** is dominated by the exchange contribution and, significantly, also shows that whereas the dipolar coupling values in both complexes are similar (*J*_dip_ ≈ –1.1 cm^–1^) the exchange couplings are significantly larger in **5-Dy** (Table 2). These observations are in agreement with the slower increase in the magnetization for **5-Dy**. The differing *J*-values for each pair of exchange-coupled dysprosium ions is a consequence of the fact they are not related by crystallographic symmetry.

**Table 2:** Exchange interactions (cm^–1^) between dysprosium ions in **4-Dy**, **5-Dy** and **6-Dy**

			*J*_dip_	*J*_ex_	*J*_tot_
**4-Dy**	Dy1–Dy2		–1.08	–3.99	–5.07
	Dy1–Dy3		–1.15	–5.72	–6.87
	Dy2–Dy3		–1.08	–3.84	–4.92
**5-Dy**	Dy1–Dy2		–1.12	–5.49	–6.61
	Dy1–Dy3		–1.11	–6.67	–7.78
	Dy2–Dy3		–1.09	–8.67	–9.76
**6-Dy**	Dy1–Dy2		–1.15	–3.61	–4.76
	Dy1–Dy3		–1.21	–3.57	–4.78
	Dy2–Dy3		–1.17	–3.85	–5.02

The exchange coupling values in Table 2 contrast markedly to those obtained for other polymetallic dysprosium complexes studied by theoretical methods, the vast majority of which feature bridging O-donor ligands with the overall exchange being dominated by the dipolar contribution.^19^,29a,^39^ The exchange interactions in O-bridged polymetallic lanthanide complexes is expected to be weaker than for arsenic-donor ligands, hence our study suggests that heavy p-block donor ligands could have an important role to play in enhancing exchange interactions in lanthanide molecular magnets. Some oxygen-bridged triangular dysprosium complexes have attracted attention in recent years owing to their toroidal magnetic moments and non-magnetic ground states,^40^ however these materials do not show the unusual magnetization behaviour observed in **4-Dy** and **5-Dy**. In the case of the selenolate-bridged complex **6-Dy**, although the exchange is once again the dominant contribution, the impact on the field-dependence of the magnetization is apparently too weak to be observed at 1.8 K. This can be explained on the basis of the isoelectronic relationship between the selenolate ligands **6-Dy** with the arsenide ligands in **4-Dy**, but with the exchange being slightly weaker owing to the less diffuse valence orbitals of selenium.

### Single-molecule magnetism

**Fig. 4:**
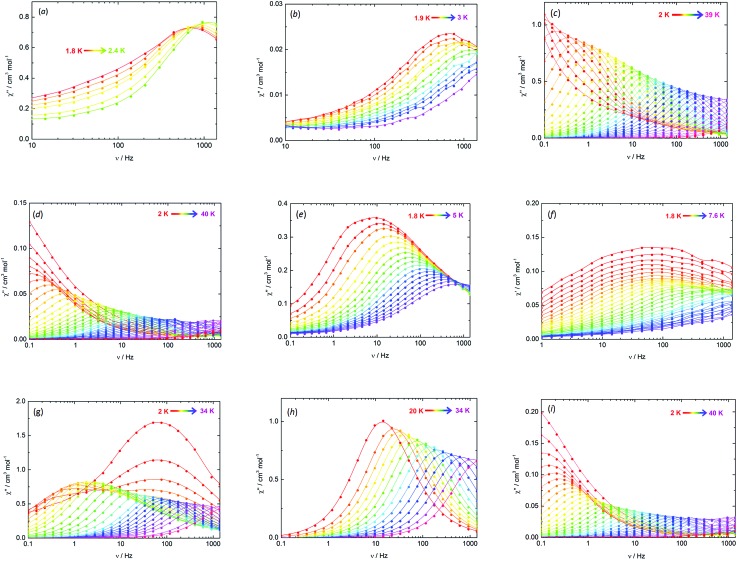
Frequency dependence of *χ*′′(*ν*) in zero applied field (unless otherwise stated) for: (a) **3-Dy** (*H*_dc_ = 1 kOe); (b) **Dy@3-Y**; (c) **4-Dy**; (d) **Dy@4-Y**; (e) **5-Dy**; (f) **Dy@5-Y**; (g) **6-Dy**; (h) **6-Dy** (*H*_dc_ = 1 kOe); (i) **Dy@6-Y**.

The SMM properties of the arsenic and selenium-ligated complexes **3-Dy**, **4-Dy**·toluene, [Li(thf)_4_]_2_[**5-Dy**]·thf and **6-Dy**·toluene were investigated through measurements of the in-phase (*χ*′) and the out-of-phase (*χ*′′) components of the magnetic susceptibility as a function of a.c. frequency (*ν*) (Fig. 4, S7–S15†). A dynamic magnetic field of *H*_ac_ = 1.55 Oe was used for each measurement. In addition, to probe the effects of exchange interactions on the dynamic magnetic properties, all four compounds were studied under conditions of 5% magnetic dilution at the single-ion level. Dilution of **3-Dy** into a lattice of **3-Y** (ref. 35) was achieved by adding MesAsH_2_ to a 1 : 20 mixture of [Cp′_3_Dy] and [Cp′_3_Y], resulting in the formation of [(Cp′_3_Dy_0.05_Y_0.95_)(AsH_2_Mes)] (**Dy@3-Y**). To obtain [(Cp′_2_Dy)(Cp′_2_Y)_2_{μ-As(H)Mes}]·toluene dispersed in a matrix of [(Cp′_2_Y){μ-As(H)Mes}]_3_·toluene (**4-Y**·toluene), denoted as **Dy@4-Y**, the deprotonation of **Dy@3-Y** was carried out in the manner used for the synthesis of **4-Dy**·toluene (Scheme 1). Similarly, [Li(thf)_4_]_2_[(η^5^-Cp′_2_Dy)(η^5^-Cp′_2_Y)_2_(μ_3_-AsMes)_3_Li]·thf in a matrix of [Li(thf)_4_]_2_[(η^5^-Cp′_2_Y)_3_(μ_3_-AsMes)_3_Li]·thf, denoted as **Dy@5-Y**, was synthesized by deprotonating **Dy@4-Y** also according to Scheme 1. Adding one stoichiometric equivalent of MesSeH to a 1 : 20 mixture of [Cp′_3_Dy] and [Cp′_3_Y] allowed access to [(Cp′_2_Dy)(Cp′_2_Y)_2_{μ-SeMes}]·toluene in a matrix of **6-Y**·toluene, denoted as **Dy@6-Y**. The doped materials were characterized by ICP atomic emission spectroscopy, which gave dysprosium levels of 5.0 ± 0.5% in each case.

The key parameters extracted from the a.c. susceptibility measurements are presented in Table 3. The *U*_eff_ values were determined by extracting the relaxation times, *τ*, from the *χ*′′(*ν*) data and using the linear sections of the relationship *τ* = *τ*_0_ exp(*U*_eff_/*k*_B_*T*) (Fig. S16†). In an applied field of *H*_dc_ = 1 kOe, broad maxima in the *χ*′′(*ν*) plots were observed for **3-Dy** at frequencies greater than approximately 1000 Hz across the temperature range 1.8–2.4 K (Fig. 4a, S7†). However, the position of the maximum moved only slightly with changes in temperature, hence a reliable *U*_eff_ value could not be extracted. In contrast, the *χ*′′(*ν*) data for **Dy@3-Y** in a 1 kOe field revealed field-induced slow relaxation of the magnetization with a very small barrier of *U*_eff_ = 8(1) cm^–1^ and *τ*_0_ = 1.25 × 10^–6^ s (Fig. 4b and S8†). In contrast to **3-Dy**, the SMM properties of **4-Dy** and **Dy@4-Y** are much more pronounced, with the *χ*′′(*ν*) plots showing strongly temperature-dependent maxima in the ranges 2–33 K and 2–34 K, respectively, in zero d.c. field (Fig. 4c, d, S9 and S10†). The anisotropy barrier for **4-Dy** was determined to be *U*_eff_ = 256(5) cm^–1^ with *τ*_0_ = 2.01 × 10^–9^ s, and upon dilution the barrier increases markedly to *U*_eff_ = 301(9) cm^–1^ with *τ*_0_ = 4.77 × 10^–10^ s.

**Table 3:** Anisotropy barriers, pre-exponential factors for the arsenic- and selenium-ligated dysprosium SMMs^*a*^

	*U*_eff_/cm^–1^	*τ*_0_/s
**3-Dy**	—	—
**Dy@3-Y**	8(1)	1.25 × 10^–6^
**4-Dy**	256(5)	2.01 × 10^–9^
**Dy@4-Y**	301(9)	4.77 × 10^–10^
**5-Dy**	23(2)	2.99 × 10^–7^
**Dy@5-Y**	35(2)	2.79 × 10^–8^
**6-Dy**	252(4)	8.30 × 10^–8^
**6-Dy** ^*b*^	285(4)	2.50 × 10^–8^
**Dy@6-Y**	301(7)	4.48 × 10^–10^

^*a*^Values determined in zero applied field unless otherwise stated.

^*b*^ *H*_dc_ = 1 kOe.

The arsinidene-ligated complex **5-Dy** shows characteristic SMM properties in zero d.c. field, however the maxima in the *χ*′′(*ν*) data were only observed in the temperature range 1.8–5 K (Fig. 4e and S11†) and the *U*_eff_ value of 23(2) cm^–1^ (*τ*_0_ = 2.99 × 10^–7^ s) is considerably smaller than in **4-Dy**. The SMM properties of the arsinidene-ligated system improve upon dilution in **Dy@5-Y** (Fig. 4f and S12†), giving *U*_eff_ = 35(2) cm^–1^ (*τ*_0_ = 2.79 × 10^–8^ s). The undiluted selenolate-bridged complex **6-Dy** (Fig. 4g and S13†) displayed two relaxation processes in zero d.c. field, *i.e.* a temperature-independent process below 4 K and a strongly temperature dependent process for which maxima in the *χ*′′(*ν*) data were observed up to 34 K. The anisotropy barrier for the thermal relaxation is *U*_eff_ = 252(4) cm^–1^ (*τ*_0_ = 8.30 × 10^–8^ s). The temperature independent process in **6-Dy** could be suppressed using an optimized field of *H*_dc_ = 1 kOe, which resulted in the observation of a single, thermal relaxation process with a barrier of *U*_eff_ = 285(4) cm^–1^ (*τ*_0_ = 2.50 × 10^–8^) (Fig. 4h and S14†). The impact of magnetic dilution on the selenolate-ligated dysprosium species was also to suppress the non-thermal relaxation processes, such that maxima in the *χ*′′(*ν*) data were observed in the temperature range 2–34 K, resulting in a barrier of *U*_eff_ = 301(7) cm^–1^ (*τ*_0_ = 4.48 × 10^–10^ s) (Fig. 4i and S15†).

Semi-circular Cole–Cole plots of *χ*′ *vs. χ*′′ were obtained and the data were fitted using a generalized Debye model with the following *α* parameters: *α* = 0.20–0.23 for **Dy@3-Y** (Fig. S17†); *α* = 0.10–0.27 for **4-Dy** and *α* = 0.08–0.39 for **Dy@4-Y** (Fig. S18†); *α* = 0.47–0.53 for **5-Dy** (Fig. S19†); *α* = 0.10–0.13 for **6-Dy** in zero field (Fig. S20†). The *α* parameters for all but one SMM imply a narrow distribution of relaxation times. Although the *α* parameters for **5-Dy** indicate a relatively wide range of relaxation times, this is not without precedent for lanthanide SMMs^10^ and can be explained by the fact that the dysprosium centres are not symmetry-related and hence are likely to be affected in different ways by the different local environments (see also Table 4 for the calculated energy spectra of the individual energy spectra on each Dy^3+^ centre).

**Table 4:** Energies (cm^–1^) of the lowest-lying Kramers doublets (KDs) of the individual Dy^3+^ centres in **3-Dy**, **4-Dy**, **5-Dy** and **6-Dy**

KD	**3-Dy**	**4-Dy**			**5-Dy**			**6-Dy**		
		Dy1	Dy2	Dy3	Dy1	Dy2	Dy3	Dy1	Dy2	Dy3
1	0.0	0.0	0.0	0.0	0.0	0.0	0.0	0.0	0.0	0.0
2	43.9	143.9	142.3	146.1	102.5	72.5	75.6	150.3	140.4	152.7
3	105.3	299.7	310.6	300.2	135.3	122.1	102.2	313.5	300.6	311.5
4	290.3	384.5	400.5	386.7	151.0	137.3	147.9	404.3	387.8	400.7
5	351.3	402.2	412.6	441.6	177.9	170.0	155.4	448.7	428.8	458.6
6	411.6	441.5	460.4	481.7	194.1	183.6	171.0	479.0	457.7	480.6
7	480.7	476.2	504.2	510.3	240.4	211.0	221.8	518.1	495.9	514.1
8	626.4	609.4	649.8	593.7	305.3	282.0	247.4	603.3	576.4	592.0

**Fig. 5:**
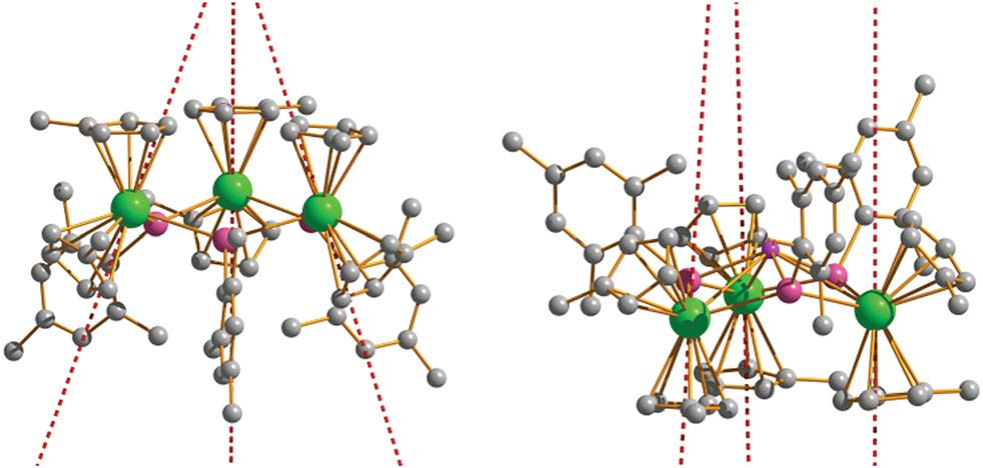
Orientation of the main magnetic axes in the ground Kramers doublets of **4-Dy** (left) and **5-Dy** (right). Dy = green, arsenic = purple, lithium = pink, carbon = grey.

Having determined the anisotropy barriers for **4-Dy**, **5-Dy**, **6-Dy** and their dilute analogues, our next aim was to identify any general trends in the magnetic properties and use this to develop magneto-structural correlations. The similar molecular structures of **4-Dy** and **6-Dy** are reflected in their calculated electronic structures. *Ab initio* calculations show that the main magnetic axes in the ground Kramers' doublets of **4-Dy** and **6-Dy** are oriented in very similar directions. In the case of **4-Dy**, the three axes are oriented at angles of 66.9–67.6° relative to the Dy_3_ plane, and in **6-Dy** they are oriented at angles of 70.8–72.1° (Fig. 5 and S26†). The magnetic axes in **5-Dy** have slightly different orientations and pass through the centre of one [Cp′]^–^ ligand, almost perpendicular (85.0–86.9°) to the Dy_3_ planes (Fig. 5). Comparing **4-Dy** and **6-Dy**, which are isoelectronic and contain formally mono-anionic arsenide and selenolate ligands, respectively, their *U*_eff_ values are essentially the same. The similar anisotropy barriers can be explained by the close similarities in their calculated low-lying energy spectra (Table 4) and *g*-tensors (Table 5). The only significant difference in the molecular structures of **4-Dy** and **6-Dy** are the Dy–E bond distances (E = As, Se), which are on average 0.079 Å shorter in **6-Dy**. While such a large difference in the metal–ligand bond distances might be expected to generate a different crystal field due to different covalent contributions to the bonding, this is likely to be moderated by the different electronegativities of the two elements, which is greater in the case of selenium (2.18 *vs.* 2.55 on the Pauling scale).^41^

**Table 5:** Calculated *g*-tensors of the ground KDs and first-excited KDs for the individual Dy^3+^ centres in **3-Dy**, **4-Dy**, **5-Dy** and **6-Dy**

		KD	*g*_*x*_	*g*_*y*_	*g*_*z*_	∠*g*_*z*1_,*g*_*z*2_^*a*^	∠*g*_*z*1_,Dy_3_^*b*^
**3-Dy**	Dy1	1	1.03	6.90	13.92	90.2	
		2	0.65	2.93	6.36		
**4-Dy**	Dy1	1	6.4 × 10^–5^	9.3 × 10^–5^	19.53	3.4	67.4
		2	3.9 × 10^–4^	4.7 × 10^–4^	17.08		
	Dy2	1	1.2 × 10^–4^	1.7 × 10^–4^	19.55	1.6	67.6
		2	4.2 × 10^–4^	5.8 × 10^–4^	17.04		
	Dy3	1	3.3 × 10^–4^	4.5 × 10^–4^	19.60	2.9	66.9
		2	3.2 × 10^–3^	3.8 × 10^–3^	17.13		
**5-Dy**	Dy1	1	3.3 × 10^–3^	4.5 × 10^–3^	19.62	44.7	85.0
		2	4.5 × 10^–2^	6.0 × 10^–2^	18.82		
	Dy2	1	3.8 × 10^–3^	8.1 × 10^–3^	19.25	35.1	86.9
		2	3.8 × 10^–2^	7.6 × 10^–2^	18.22		
	Dy3	1	2.7 × 10^–3^	3.5 × 10^–3^	19.35	42.0	85.2
		2	0.12	0.18	18.14		
**6-Dy**	Dy1	1	1.1 × 10^–4^	1.5 × 10^–4^	19.60	7.1	72.1
		2	8.9 × 10^–4^	1.1 × 10^–3^	17.16		
	Dy2	1	1.9 × 10^–4^	2.2 × 10^–4^	19.49	6.8	71.4
		2	8.8 × 10^–4^	1.1 × 10^–3^	17.12		
	Dy3	1	1 × 10^–6^	1.6 × 10^–5^	19.61	3.4	70.8
		2	2.4 × 10^–4^	3.6 × 10^–4^	17.14		

^*a*^∠*g*_*z*1_,*g*_*z*2_ angle formed at the intersection of the main magnetic axes in the ground KD and first-excited KD.

^*b*^∠*g*_*z*1_,Dy_3_ angle formed between the main magnetic axes in the ground KD and the Dy_3_ plane.

The Dy–As distances in **5-Dy** are shorter by an average of 0.128 Å than those in **4-Dy**. Extending the argument used to compare **4-Dy** and **6-Dy**, the closer proximity of the arsenic atoms in **5-Dy**, combined with the more diffuse orbitals on the donor atoms, is likely to produce a stronger crystal field as a consequence of enhanced covalent character in the metal–ligand bonds. This argument is consistent with the smaller overall splitting of the eight lowest-lying Kramers' doublets in **5-Dy** (Table 4), and can account for the much smaller *U*_eff_ value in this complex and in its dilute analogue **Dy@5-Y**.

To the best of our knowledge, the current record anisotropy barrier for any type of SMM is the value of *U*_eff_ = 652 cm^–1^ found in a heteroleptic terbium(iii) phthalocyanine complex of the type [TbPcPc′].^42^ The largest anisotropy barriers in polymetallic dysprosium SMMs are *U*_eff_ = 481 cm^–1^ and 585 cm^–1^ for magnetically non-dilute and dilute versions of a high-symmetry alkoxide cage complex.^43^ The largest barrier in a metallocene SMM is *U*_eff_ = 330 cm^–1^ in the magnetically dilute version of [(Cp*_2_Dy)(μ-BPh_4_)] (Cp* = pentamethylcyclopentadienide).^30^ In the broader context of anisotropy barriers determined for lanthanide SMMs in zero d.c. field, the *U*_eff_ values of **4-Dy**, **6-Dy** and their magnetically dilute analogues are in the region of 250–300 cm^–1^, placing them amongst the largest barriers yet reported.

The calculated energies of the eight lowest-lying Kramers doublets and the associated *g*-tensors on the individual dysprosium centres provide further insight into the magnetic relaxation in the arsenic- and selenium-ligated SMMs (Tables 4, 5, S4–S10†). In the case of **3-Dy**, the absence of zero-field SMM behaviour can be explained in terms of the weak axial character of the ground Kramers doublet, which possesses *g*_*x*_ = 1.03, *g*_*y*_ = 6.90 and *g*_*z*_ = 13.92, *i.e.* the deviations from the Ising limit of a ground state with predominant |*m*_*J*_| = 15/2 character are significant. Thus, the relaxation in **3-Dy** is likely to be dominated by efficient Raman and direct processes and/or QTM within the ground Kramers doublet. In the case of the **4-Dy**, the ground Kramers doublet and the first- and second-excited Kramers doublets show considerable axial character, and correspond to states with predominant |*m*_*J*_| = 15/2, 13/2 and 11/2 character, respectively. Furthermore, the calculations reveal that the main magnetic axes in the two lowest-lying Kramers doublets are essentially co-linear, with the axes in the first-excited doublets being oriented at angles of 1.6–3.4° relative to the ground doublet (Table 5), respectively, which is the requirement for thermal relaxation *via* the second-excited Kramers doublet. The calculated energy gaps from the ground doublets of the individual dysprosium centres in **4-Dy** to the second-excited doublets are 300–310 cm^–1^, which agrees extremely well with the observed barrier in **Dy@4-Y** of *U*_eff_ = 301(9) cm^–1^. The similarities in the a.c. susceptibility data for **4-Dy** and **6-Dy**, and their diluted analogues, are reflected in their calculated electronic structure, such that thermal relaxation in **6-Dy** should also occur *via* the second-excited Kramers doublets. The thermal barrier of *U*_eff_ = 301(7) cm^–1^ determined for **Dy@6-Y** again gives excellent agreement with the calculated energy gaps of 300–313 cm^–1^. Although relaxation *via* Kramers doublets that lie above the first-excited doublet has been previously described,^43^,^44^ such processes are still uncommon.

The ground and first-excited Kramers doublets in the arsinidene-ligated SMM **5-Dy** have strong axial character, however the *g*-tensors of first-excited doublets have much larger transverse components and they are oriented at angles of 35.1–44.7° relative to the ground doublet. These data suggest that the thermal relaxation process in **5-Dy** and **Dy@5-Y** involves excitation only to the first-excited doublet, in contrast to **4-Dy** and **6-Dy**. The corresponding calculated energy gaps are 72 cm^–1^, 76 cm^–1^ and 102 cm^–1^, which are much larger than the observed barrier of *U*_eff_ = 35(2) cm^–1^ in **Dy@5-Y**, implying that non-thermal relaxation processes such as QTM are significant in the arsinidene-ligated SMMs.

### Magnetic hysteresis

The importance of magnetic hysteresis in nanoscale magnetic materials stems from their potential applications in information storage devices,^45^ although in most SMMs the hysteresis loops are narrow (if observed at all), the coercive fields and remanent magnetization are very weak, and liquid-helium temperatures are essential.^1^–^5^ The most impressive hysteresis in an SMM found to date was a very large coercive field of approximately almost 5 Tesla at 11 K, which was observed in a di-terbium radical-bridged complex.^46^ Aside from the potential applications of SMMs, there is considerable fundamental interest in understanding how coordination chemistry can be used to influence the magnetic hysteresis properties and, in this context, 4p element donor ligands have not previously been studied.

**Fig. 6:**
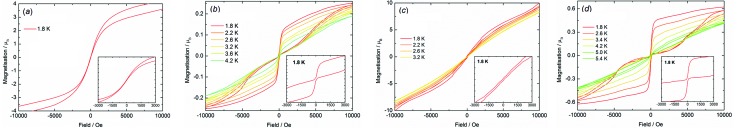
*M*(*H*) hysteresis for: (a) **3-Dy** (1.90 mT s^–1^); (b) **Dy@3-Y** (3.06 mT s^–1^); (c) **4-Dy** (2.87 mT s^–1^); (d) **Dy@4-Y** (3.14 mT s^–1^).

Hysteresis measurements on all compounds were made using fields in the range ±5 T. The undiluted compounds **3-Dy** (Fig. 6a), **4-Dy** (Fig. 6c), [Li(thf)_4_]_2_[**5-Dy**] thf (Fig. S21†) and **6-Dy** (Fig. S22†) exhibit S-shaped *M*(*H*) hysteresis curves at 1.8 K, with only very slight opening of the loops. In contrast, the hysteresis properties of **Dy@3-Y** (Fig. 6b), **Dy@4-Y** (Fig. 6d) and **Dy@6-Dy** (Fig. S22†) are markedly different to their undiluted analogues, producing open hysteresis loops at temperatures in the range 1.8–4.2 K, 1.8–5.4 K and 1.8–4.7 K, respectively, with average scan rates of 3.1 mT s^–1^, 3.8 mT s^–1^ and 3.1 mT s^–1^. At 1.8 K, the value of the magnetization in **Dy@3-Y**, **Dy@4-Y** and **Dy@6-Y** gradually decreases with decreasing field and then experiences a sharp drop close to zero field owing to rapid QTM. Hence, the hysteresis features vanishingly small coercivity and remanent magnetization. As the measurement temperature increases the loops gradually close. Although the hysteresis loops observed for the magnetically dilute compounds are similar in appearance to those observed previously in some dysprosium SMMs,^1^–^3^ the change in the property relative to their magnetically non-dilute systems is remarkable. This is particularly so for **Dy@3-Y** in light of the fact that an anisotropy barrier of only 8 cm^–1^ was recorded in zero field, indicating that intermolecular dipolar interactions are significant in the arsine-ligated systems.

## Conclusions

The dysprosium-arsine complex **3-Dy** is a precursor to the arsenide-bridged complex **4-Dy**, which can itself be deprotonated to give [Li(thf)_4_]_2_[**5-Dy**]. Complex **5-Dy** is the first lanthanide arsinidene complex. Deprotonation of mesitylselenol by Cp′_3_M (M = Y or Dy) produced the selenolate-bridged complexes **6-Y** and **6-Dy**, the structures of which are very similar to that of **4-Dy** and closely related to that of **5-Dy**. The field dependence of the magnetization in **4-Dy** and **5-Dy** at 1.8 K show distinct plateaus around fields of approximately 10 kOe, which was attributed to switching of the magnetic ground state from one with dominant antiferromagnetic exchange to one with ferromagnetic exchange. The plateaus in the *M*(*H*) data are extremely unusual for a polymetallic lanthanide complex, and were attributed to the influence of strong exchange interactions. In particular, the dominance of the arsenic-mediated exchange over the dipolar exchange between the dysprosium centres is important in these systems. The slower increase in the magnetization with increasing field observed for **5-Dy** relative to **4-Dy** shows that different types of arsenic ligand can influence the magnetic properties of lanthanide complexes.

Complexes **4-Dy** and **6-Dy** are SMMs with energy barriers in the region of *U*_eff_ = 250 cm^–1^ in zero field. A much smaller barrier of *U*_eff_ = 23 cm^–1^ was determined for **5-Dy** in zero field. The energy barriers of all three trimetallic complexes increase upon magnetic dilution, with the values of *U*_eff_ = 301 cm^–1^ for **4-Dy** and **6-Dy** being amongst the highest yet reported in zero d.c. field. The much smaller barriers determined for the **5-Dy** and **Dy@5-Y** are due to the stronger crystal field generated by the arsinidene ligands, which in turn can be rationalized in terms of small-but-significant increases in the covalent contribution to the metal–ligand bonding. Whereas all magnetically undiluted compounds show narrow *M*(*H*) hysteresis loops at 1.8 K, 5% doping at the single-ion level produced butterfly-shaped hysteresis loops up 4.2 K for **Dy@3-Y**, up to 5.4 K for **Dy@4-Y** and up to 4.7 K for **Dy@6-Y**.

The use of ligands with heavier p-block elements as the donor atoms, potentially including metallic donor atoms, could prove to be an effective strategy for enhancing the properties of single-molecule magnets.

## Supplementary Material

Supplementary information

Crystal structure data
