# Supplementary material for: Magneto-structural correlations in arsenic- and selenium-ligated dysprosium single-molecule magnets
Source: Chem Sci. 2015 Dec 15;7(3):2128–37. doi: 10.1039/c5sc03755g (PMC5968533; doi:10.1039/c5sc03755g)
Supplement: Supplementary file 1 [file SC-007-C5SC03755G-s001.pdf]

Electronic Supplementary Material (ESI) for Chemical Science.
This journal is © The Royal Society of Chemistry 2015

Magneto-Structural Correlations in Arsenic- and Selenium-Ligated Dysprosium Single-Molecule Magnets

Thomas Pugh,[1] Veacheslav Vieru,[2]
Liviu F. Chibotaru[2] and Richard A. Layfield[1]*

[1] School of Chemistry, The University of Manchester, Oxford Road, Manchester, M13 9PL, U.K.

[2] Theory of Nanomaterials Group, Katholieke Universiteit Leuven, Celestijenlaan 200F, 3001 Heverlee, Belgium.

* Richard.Layfield@manchester.ac.uk

Table of Contents

1. Synthetic details pp 2-3
2. NMR spectroscopy of **4-Y** and IR spectroscopy pp 4-5
3. X-ray crystallography pp 5-6
4. Magnetic property measurements pp 7-12
5. Computational details pp 13-21
6. References pp 21

General Synthetic Details. All manipulations were performed under an atmosphere of dry, oxygen-free argon, using either standard Schlenk techniques or an argon-filled glove box. Toluene and toluene-d_8 were dried by refluxing over potassium and distilled. All solvents were stored over activated 4 Å molecular sieves or a potassium mirror and freeze-thaw degassed prior to use. Anhydrous rare earth chlorides (99.99% purity), *n*-butyllithium (1.6 M in hexanes), arsenic(III) chloride, lithium dimethylamide, lithium aluminium hydride, mesitylmagnesium bromide (1.0 M in THF) and di-methylcyclopentadiene (95%) were purchased from Sigma-Aldrich. Mesitylarsine[1] and rare-earth *tris*-methylcyclopentadienide complexes were synthesised according to literature procedures.[2] Mesitylselenol was prepared analogously to phenylselenol.[3,4] Elemental analyses were carried out at London Metropolitan University, U.K. Infrared spectra were recorded as Nujol mulls in KBr discs on a Shimadzu IRAffinity-1S FT-IR spectrophotometer. X-ray diffraction data were collected on an Oxford Instruments XCalibur2 diffractometer or an Agilent SuperNova, using MoKα radiation, or a Bruker APEX-II diffractometer, using CuKα radiation. NMR spectra were acquired on a Bruker Avance-III 400 MHz spectrometer.

MesSeH. Selenium powder (0.21 g, 2.63 mmol) was added to a Schlenk flask containing MesMgBr (1.0 M in Et_2O, 2.80 ml, 2.80 mmol) at ambient temperature over a period of 10 minutes; once addition was complete the reaction mixture was stirred for 1 hour. Crushed ice (5 g) was then added, followed by dropwise addition of HCl (36 % in H_2O, 0.36 ml). The reaction mixture was then filtered into a separating funnel and the product extracted into Et_2O (3 × 10 ml). The combined extracts were dried over $CaCl_2$ and the Et_2O was removed *in vacuo*. The residual yellow oil was distilled (60°C at 4 Torr) to yield a colourless oil (0.35 g, 67%). The ^1H NMR chemical shifts of the product were identical to those previously reported.[4]

[Cp′₃Dy(AsH₂Mes)] (3-Dy). $MesAsH_2$ (0.10 g, 0.50 mmol) in toluene (2 ml) was added to a solution of Cp'_3Dy (0.20 g, 0.50 mmol) in toluene (4 ml) at room temperature, and the reaction mixture was stirred for one hour. The resulting pale-yellow solution was filtered, concentrated and stored at –30°C overnight, which resulted in the formation of **3-Dy** as colourless crystals (0.25 g, 83% isolated yield). Elemental analysis, found/% (calculated/%) for **3-Dy**: C, 54.37 (54.41); H, 5.68 (5.75). Infrared spectrum: $\tilde{\nu}_{As-H}$ = 2139 cm^{-1}.

[(Cp′₂Dy){*μ*-AsH)Mes}]₃·toluene (4-Dy·toluene). ^{n}BuLi (1.6 M in hexanes, 0.32 ml, 0.50 mmol) was added to a solution of **3-Dy** (0.30 g, 0.50 mmol) in toluene (20 ml) at –78°C, and the reaction mixture was stirred for one hour. The reaction was then slowly warmed to room temperature overnight, after which time a pale yellow solution and a precipitate had formed. The resulting pale-yellow solution was filtered and concentrated, which resulted in the formation of a pale yellow precipitate. The precipitate was re-dissolved and the solution stored at –30°C overnight, which resulted in the formation of **4-Dy**·toluene as colourless crystalline blocks (0.18 g, 66%). Elemental analysis, found/% (calculated/%) for **4-Dy**·toluene: C, 51.31 (51.28); H, 5.12 (5.29). Infrared spectrum: $\tilde{\nu}_{As-H}$ = 2110, 2153 cm^{-1}.

[(Cp′₂Dy)₃(*μ*-AsMes)₃Li][Li(thf)₄]₂·thf ([5-Dy][Li(thf)₄]₂·thf). A solution of **4-Dy**·toluene (0.16 g, 0.10 mmol) in thf (7 ml) was cooled to –20°C and ^{n}BuLi (1.6 M in hexanes, 0.29 ml, 0.18 mmol) was added drop-wise. After stirring at –10 °C for 30 minutes, the reaction was warmed to room temperature over 2 hours, after which time a bright orange colour had developed. The solution was concentrated until copious amounts of precipitate had formed, then the precipitate was re-dissolved by gentle heating and the solution stored at +4°C. [**5-Dy**][Li(thf)$_4$]$_2$·thf formed as orange blocks (0.17 g, 77%). Elemental analysis, found/% (calculated/%) for [**5-Dy**][Li(thf)$_4$]$_2$·thf: C, 53.81 (53.70); H, 6.62 (6.69).

[(Cp′₂Dy){*μ*-SeMes}]₃·toluene (6-Dy·toluene). MesSeH (0.5 M solution in toluene, 1 ml, 0.50 mmol) was added to a solution of Cp'_3Dy (0.20 g, 0.50 mmol) in toluene (10 ml) at –78°C, and the reaction mixture was stirred for one hour. The reaction was then slowly warmed to room temperature overnight, after which time a pale-yellow solution had formed. The resulting pale-yellow solution was concentrated, which resulted in the formation of a precipitate. The precipitate was re-dissolved and the solution stored at –30°C overnight, which resulted in the formation of **6-Dy**·toluene as colourless crystalline blocks (0.25 g, 91%). Elemental analysis, found/% (calculated/%) for **6-Dy**·toluene: C, 50.88 (50.99); H, 5.14 (5.07).

[(Cp′₂Y){*μ*-SeMes}]₃·toluene (6-Y·toluene). MesSeH (0.5 M solution in toluene, 1.2 ml, 0.60 mmol) was added to a solution of Cp'_3Y (0.20 g, 0.60 mmol) in toluene (10 ml) at –78°C, and the reaction mixture was stirred for one hour. The reaction was then slowly warmed to room temperature overnight, after which time a

pale-yellow solution had formed. The resulting pale-yellow solution was concentrated, which resulted in the formation of a pale yellow precipitate. The precipitate was re-dissolved and the solution stored at –30°C overnight, which resulted in the formation of **6-Y**·toluene as colourless crystalline blocks (0.23 g, 79%). Elemental analysis, found/% (calculated/%) for **6-Y**·toluene: C, 58.71 (58.88); H, 5.95 (5.86). ^{1}H NMR (toluene-d$_8$, 298.15 K, δ/ppm): 6.92 (s, 6H, mesityl CH); 6.06, 6.03 (br s, 24H, Cp' CH); 2.79 (s, 18H, mesityl *ortho*-CH_3); 2.11 (s, 9H, mesityl *para*-CH_3); 2.01 (s, 18H, Cp' CH_3). ^{13}C NMR (toluene-d$_8$, 298.15 K, *δ*/ppm): 141.57 (*ipso*-CSe); 136.22 (*ortho*-CMe); 134.95 (*para*-CMe), 128.63 (Mes CH), 122.99 (Cp' CMe), 115.05, 112.44 (Cp'CH), 28.12 (*ortho*-Me), 21.21 (*para*-Me), 15.40 (Cp′-Me).

Doped sample Dy@3-Y. The dilution was achieved by addition of $MesAsH_2$ (0.20 g, 1.00 mmol) a solution of Cp'_3Dy (0.02 g, 0.05 mmol) and Cp'_3Y (0.31 g, 0.95 mmol) in toluene (4 ml) at room temperature, and the reaction mixture was stirred for one hour. The resulting pale-yellow solution was filtered, concentrated and stored at –30°C overnight, which resulted in the formation of **Dy@3-Y** as colourless crystals (0.38 g, 72%).

Doped sample Dy@4-Y·toluene. The dilution was achieved by deprotonation of **Dy@3-Y** (0.52 g, 1.00 mmol) in toluene (20 ml). Following the addition of ^{n}BuLi (1.6 M, 0.63 ml, 1 mmol) at –78°C and the same work-up procedure as described for **4-Dy** (see above), the doped sample was obtained as colourless crystals (0.27 g, 50%).

Doped sample [Li(thf)$_4$]$_2$[Dy@5-Y]·thf/[Li(thf)$_4$]$_2$[5-Y]·thf. The dilution was achieved by the deprotonation of **Dy@4-Y·toluene** (0.15 g, 0.10 mmol) by ^{n}BuLi (1.6 M, 0.19 ml, 0.31 mmol) at –10°C, with the workup procedure as described for **[Li(thf)$_4$]$_2$[5-Dy]**·thf (see above), the doped sample was obtained as orange blocks (0.12 g, 62%).

Doped sample Dy@6-Y·toluene. The dilution was achieved by adding MesSeH (0.5M solution in toluene, 1 ml, 0.5 mmol) to a mixture of Cp'_3Dy (0.01 g, 0.025 mmol) and Cp'_3Y (0.16 g, 0.48 mmol) in toluene (10 ml) at –78°C. Following the workup procedure as described for **6-Dy** (see above), the doped sample was obtained as colourless crystals (0.17 g, 73%).

Characterization of doped materials

The four doped materials were characterized by X-ray diffraction (Table S1): measurements of the unit cell parameters of several crystals of each were performed on an Oxford Xcaliber-2 diffractometer using Mo-Kα radiation at 100 K. The unit cell dimensions of both compounds were found to be equivalent (within 3σ) to those observed for their respective pure yttrium compounds. Accurate dysprosium/yttrium ratios were measured by inductively coupled plasma atomic emission (ICP) spectroscopy using a Thermo iCap 6300 ICP-OES instrument, which resulted in dysprosium contents of 5.0±0.5% for all doped materials.

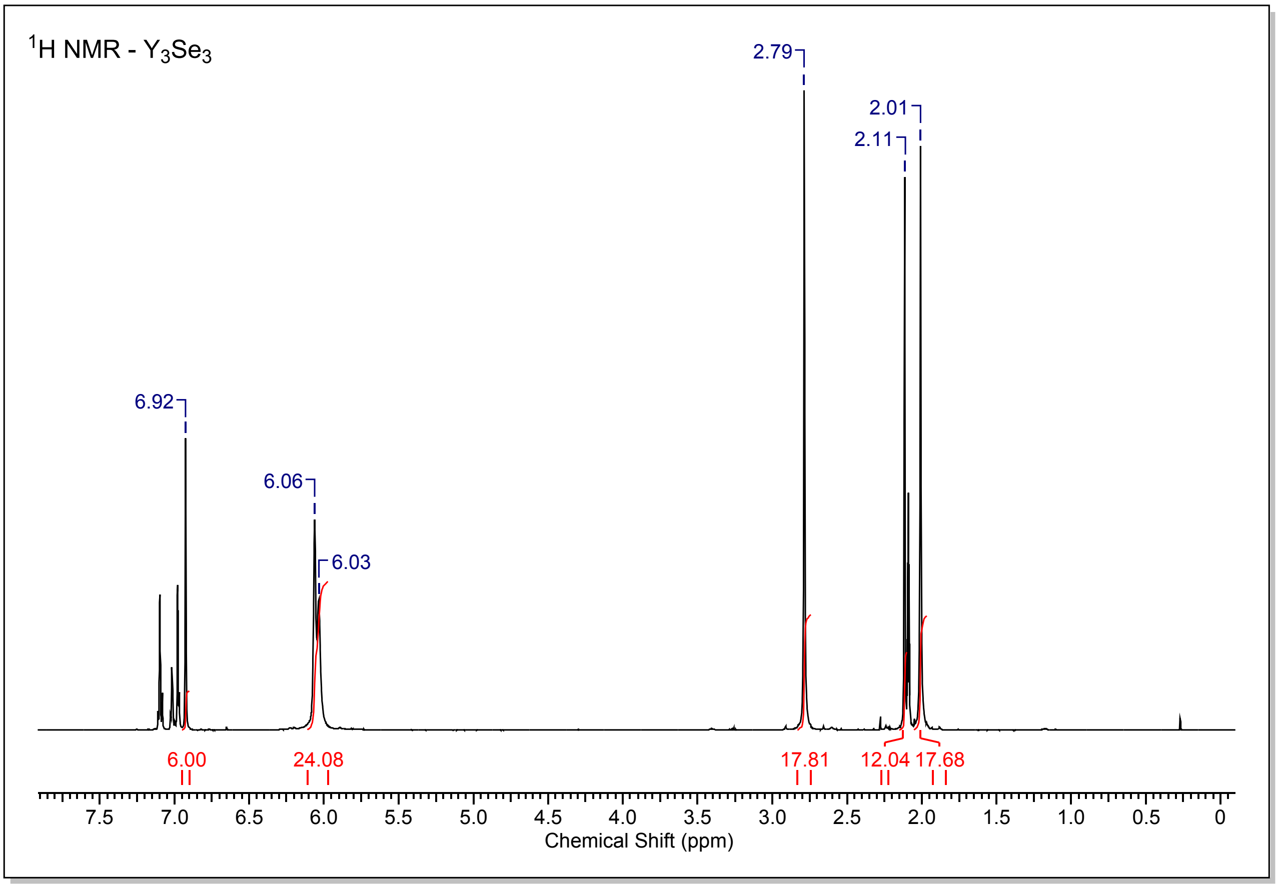

Figure S1. ^{1}H NMR spectrum of **6-Y** in toluene-D$_8$ at 298 K.

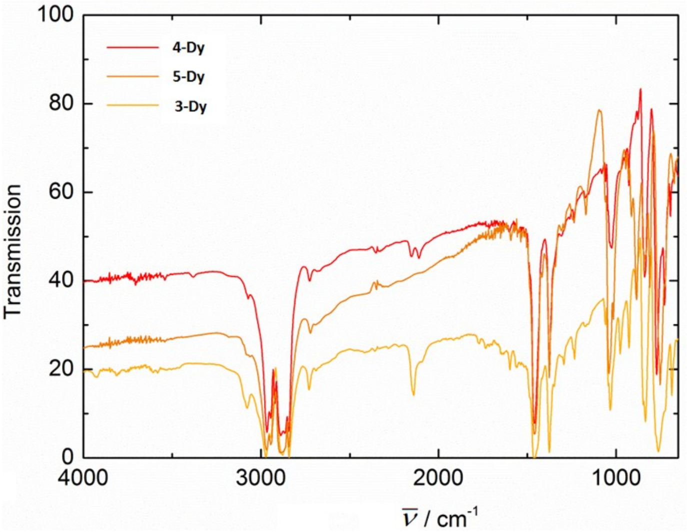

Figure S2. Infrared spectra (Nujol mulls) of **3-Dy** ($\tilde{\nu}_{As\text{–}H}$ = 2139 cm^{-1}), **4-Dy**·toluene ($\tilde{\nu}_{As\text{–}H}$ = 2110, 2153 cm^{-1}) and [Li(thf)$_4$]$_2$[**5-Dy**]·thf.

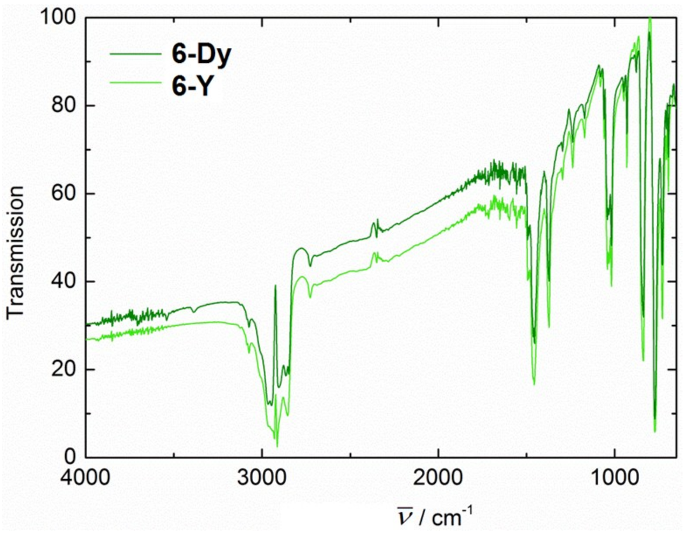

Figure S3. Infrared spectra (Nujol mulls) of **6-Dy**·toluene and **6-Y**·toluene.

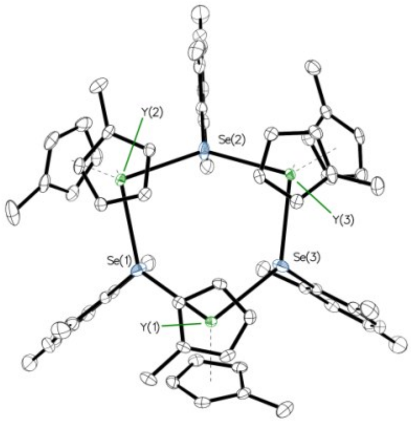

Figure S4. Thermal ellipsoid representations (50% probability) of the molecular structure of **6-Y**. For clarity, hydrogens atoms have been omitted.

Table S1. Crystal data and structure refinement details. MoKα (λ = 0.71073) and *CuKα (λ = 1.54178).

	**3-Dy**	**4-Dy·toluene**	**[Li(thf)$_4$]$_2$[5-Dy]·thf**	**6-Dy·toluene**	**6-Y·toluene***
empirical formula	$C_{27}H_{34}DyAs$	$C_{70}H_{83}Dy_3As_3$	$Dy_3As_3C_{99}H_{132}O_9Li_3$	$C_{70}H_{83}Dy_3Se_3$	$C_{70}H_{83}Y_3Se_3$
formula weight	595.96	1636.62	2214.24	1648.74	1427.97
temperature / K	100	150	150	100	100
crystal system	monoclinic	monoclinic	monoclinic	monoclinic	monoclinic
space group	$P2_1/n$	Cc	$P2_1/c$	Cc	Cc
a / Å	8.1717(3)	14.9242(3)	22.4510(4)	14.8845(13)	14.90290(10)
b / Å	24.8088(10)	18.6105(4)	15.8357(3)	18.4953(14)	18.42530(10)
c / Å	11.6477(5)	23.7155(5)	28.0477(5)	23.4214(18)	23.4230(2)
α / °	90	90	90	90	90
β / °	94.354(4)	103.461(2)	95.470(2)	103.098(8)	103.0350(10)
γ / °	90	90	90	90	90
volume / Å^3	2354.53(17)	6406.0(3)	9926.3(3)	6280.0(9)	6266.00(8)
Z	4	4	4	4	4
ρ_{calc} / mg mm^{-3}	1.681	1.697	1.482	1.744	1.514
crystal size / mm^3	0.2 × 0.2 × 0.1	0.2 × 0.15 × 0.1	0.3 × 0.2 × 0.1	0.2 × 0.1 × 0.1	0.2 × 0.1 × 0.1
2θ range/°	5.886 to 50.05	5.816 to 50.696	6.552 to 50.056	7.554 to 50.7	7.752 to 147.448
reflections collected	14983	20817	128160	37134	23361
independent reflections	4163	10263	17494	11451	9014
R(int)	0.0339	0.0358	0.0402	0.0675	0.0187
data/restraints/parameters	4163/127/332	10263/184/701	17494/401/1299	11451/74/701	9014/2/701
goodness-of-fit on F^2	1.050	1.117	1.102	1.021	1.076
final R indexes [I≥2σ(I)]	R_1 = 0.0252<br>wR_2 = 0.0516	R_1 = 0.0384<br>wR_2 = 0.0894	R_1 = 0.0303<br>wR_2 = 0.0622	R$_1$ = 0.0401<br>wR$_2$ = 0.806	R$_1$ = 0.0224<br>wR$_2$ = 0.0582
final R indexes [all data]	R_1 = 0.0302<br>wR_2 = 0.0545	R_1 = 0.0430<br>wR_2 = 0.1035	R_1 = 0.0374<br>wR_2 = 0.0648	R$_1$ = 0.0512<br>wR$_2$ = 0.0870	R$_1$ = 0.0225<br>wR$_2$ = 0.0582
largest diff. peak, hole /e.A^{-3}	0.81, –0.81	3.24, –1.96	1.24, –1.15	1.67, –0.86	1.00, –0.99
CCDC reference code	1403610	1403611	1403612	1403613	1403614

Magnetic property measurements

The magnetic properties of polycrystalline samples of **3-Dy**, **4-Dy**·toluene, $[Li(thf)_4]_2$[**5-Dy**]·thf, **6-Dy**, **Dy@3-Y**, **Dy@4-Y**·toluene, [**Dy@5-Y**]$[Li(thf)_4]_2$·thf and **Dy@6-Y**·toluene were measured using a Quantum Design MPMS-7 SQUID magnetometer at temperatures in the range 1.8-300 K. In a glove box, the polycrystalline samples were transferred to NMR tubes, restrained in eicosane and flame sealed under vacuum.

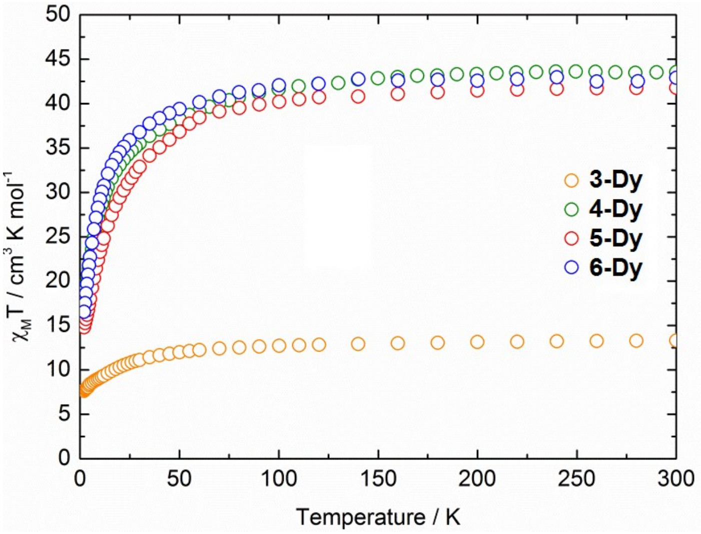

Figure S5. The product of the molar magnetic susceptibility with temperature (χ_MT) against temperature (T) for **3-Dy**, **4-Dy**·toluene, $[Li(thf)_4]_2$[**5-Dy**]·thf and **6-Dy**·toluene, collected in an applied field of 1 kOe.

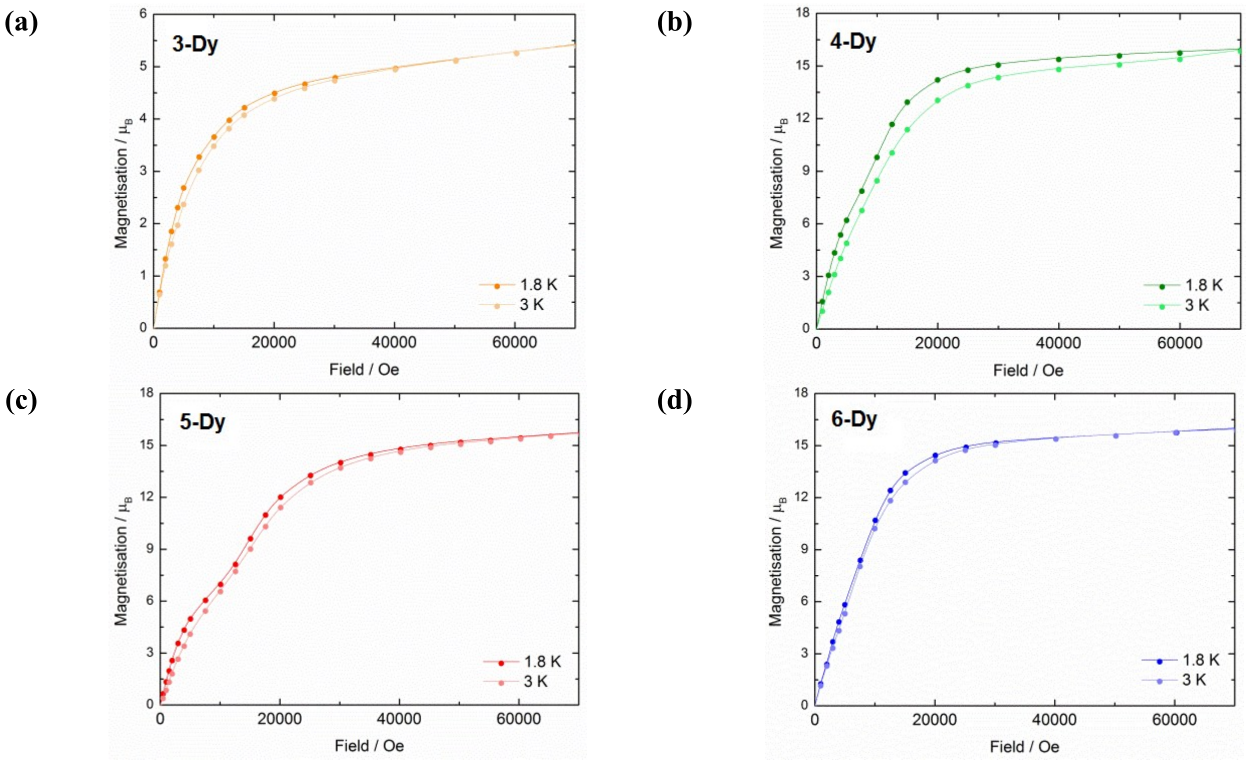

Figure S6. Field dependence of the magnetization for: (a) **3-Dy**; (b) **4-Dy**·toluene; (c) $[Li(thf)_4]_2$[**5-Dy**]·thf; (d) and **6-Dy**·toluene. Data collected at 1.8 K and 3 K.

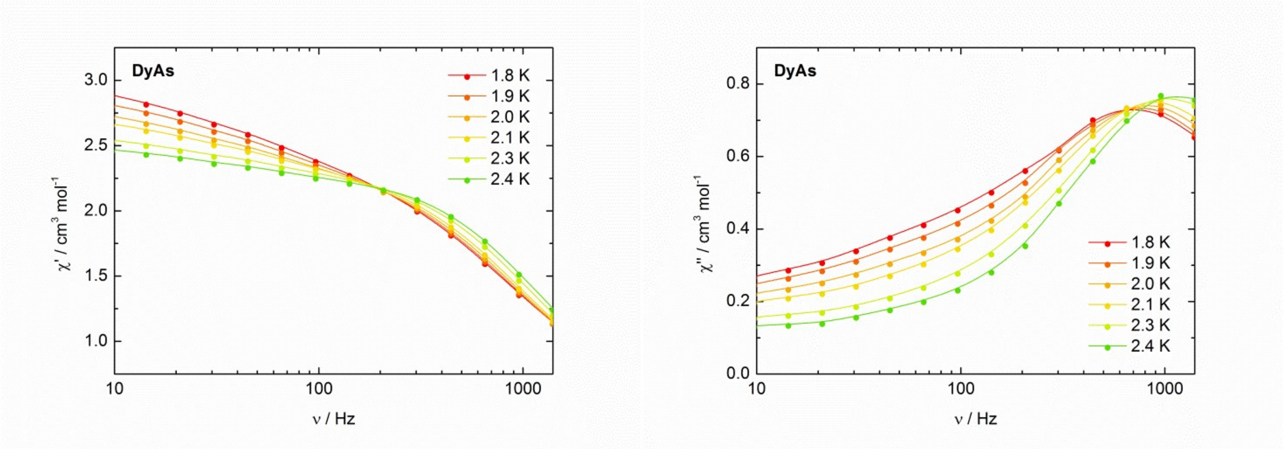

Figure S7. Frequency dependence of the in-phase (χ') and the out-of-phase (χ'') magnetic susceptibility for **3-Dy** using an oscillating field of H_{ac} = 1.55 Oe and 1 kOe applied field.

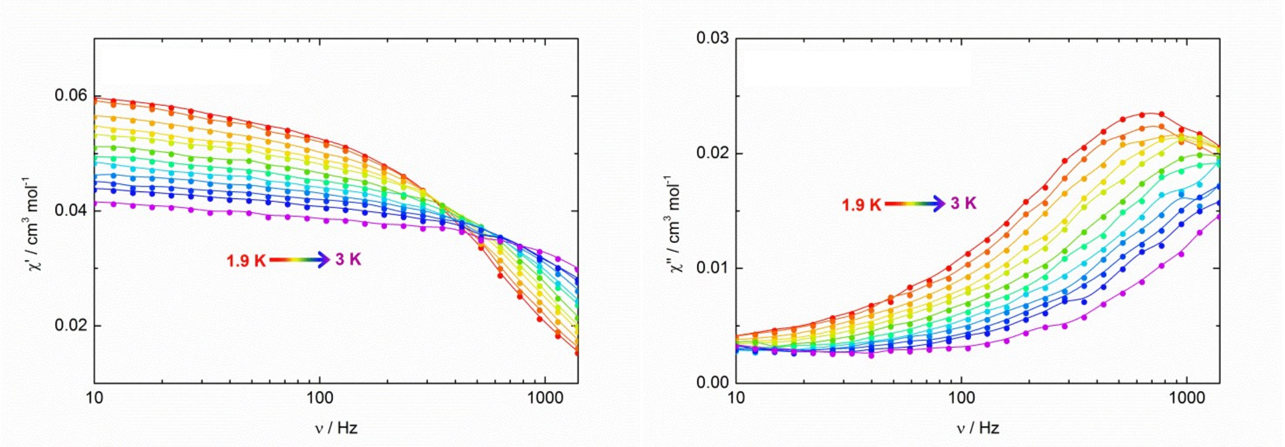

Figure S8. Frequency dependence of the in-phase (χ') and the out-of-phase (χ'') magnetic susceptibility for **Dy@3-Y** in a matrix of **3-Y** (1:20 Dy:Y). Data collected using an oscillating field of H_{ac} = 1.55 Oe and 1 kOe applied field.

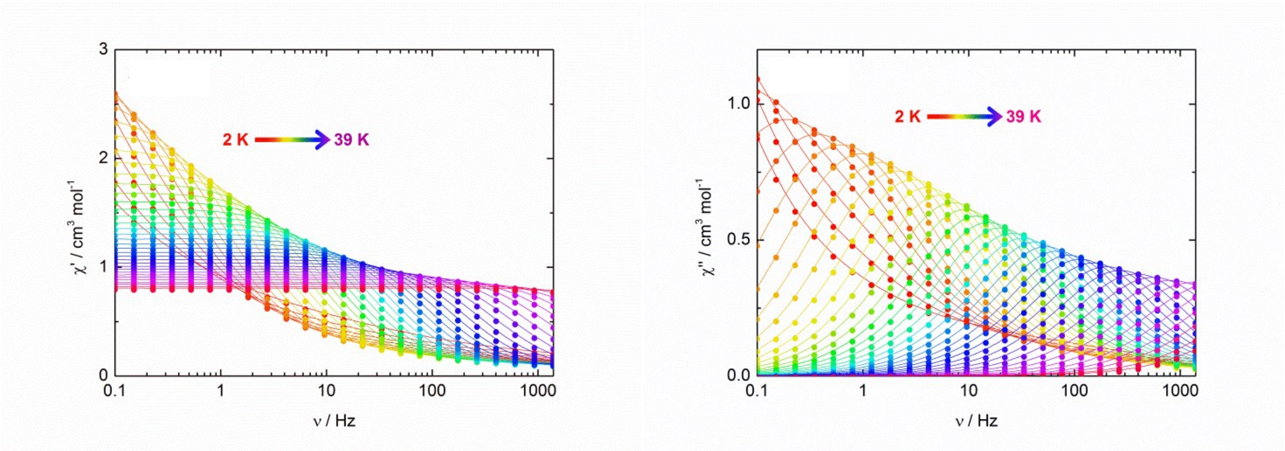

Figure S9. Frequency dependence of the in-phase (χ') magnetic susceptibility for **4-Dy**·toluene using an oscillating field of H_{ac} = 1.55 Oe and zero applied field.

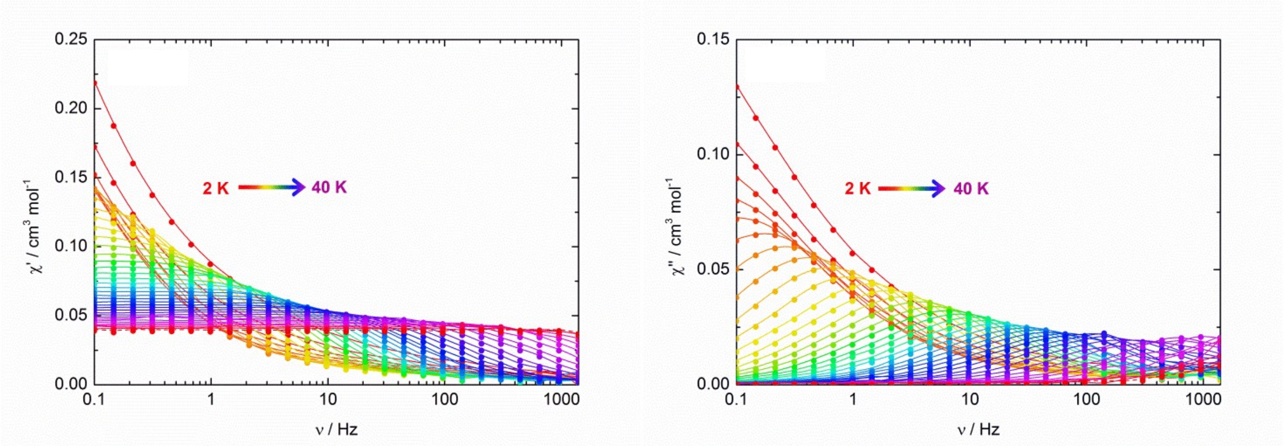

Figure S10. Frequency dependence of the in-phase (χ') and the out-of-phase (χ'') magnetic susceptibility for **Dy@4-Y**·toluene in a matrix of **4-Y**·toluene (1:20 Dy:Y). Data collected using an oscillating field of H_{ac} = 1.55 Oe and zero applied field.

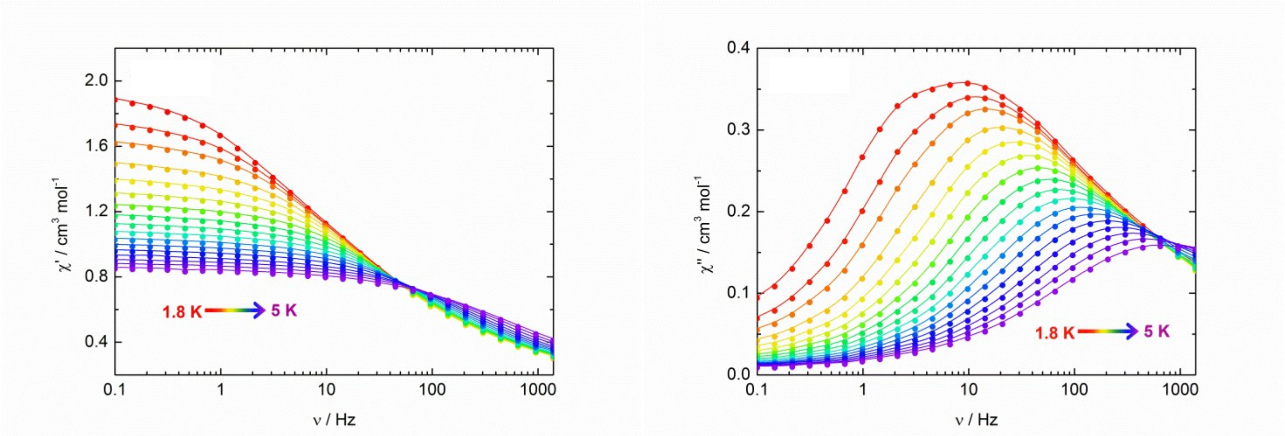

Figure S11. Frequency dependence of the in-phase (χ') magnetic susceptibility for [Li(thf)$_4$]$_2$[**5-Dy**]·thf using an oscillating field of H_{ac} = 1.55 Oe and zero applied field.

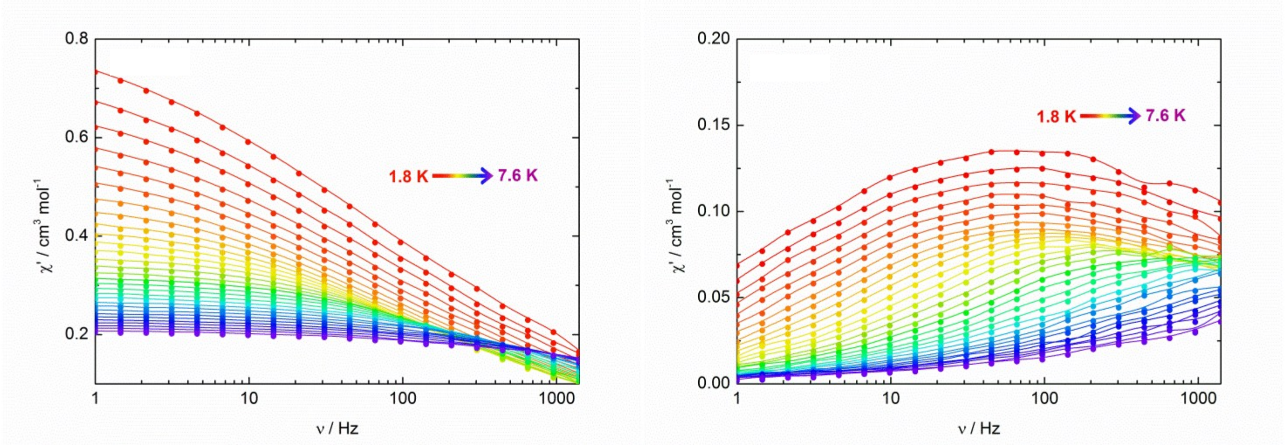

Figure S12. Frequency dependence of the in-phase (χ') and the out-of-phase (χ'') magnetic susceptibility for [Li(thf)$_4$]$_2$[**Dy@5-Y**]·thf in a matrix of [Li(thf)$_4$]$_2$[**5-Y**]·thf (1:20 Dy:Y). Data collected using an oscillating field of H_{ac} = 1.55 Oe and zero applied field.

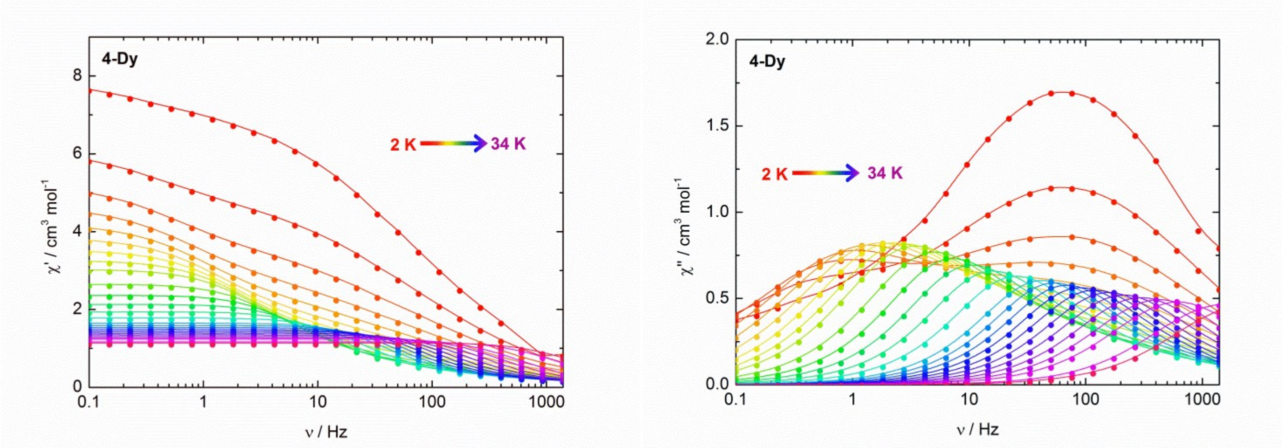

Figure S13. Frequency dependence of the in-phase (χ') magnetic susceptibility for **6-Dy**·toluene, using an oscillating field of H_{ac} = 1.55 Oe and zero applied field.

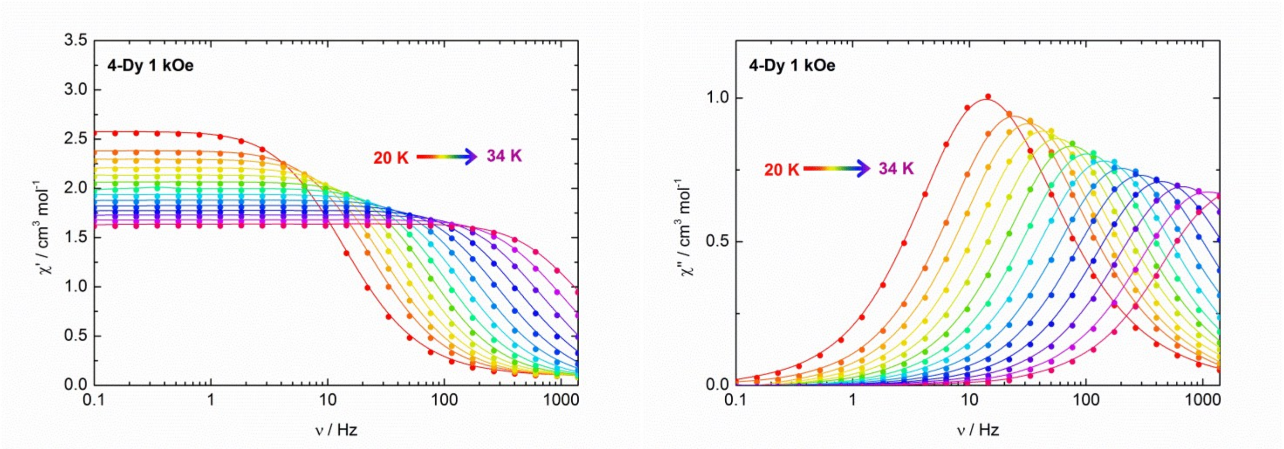

Figure S14. Frequency dependence of the in-phase (χ') magnetic susceptibility for **6-Dy**·toluene, using an oscillating field of H_{ac} = 1.55 Oe and a 1 kOe applied field.

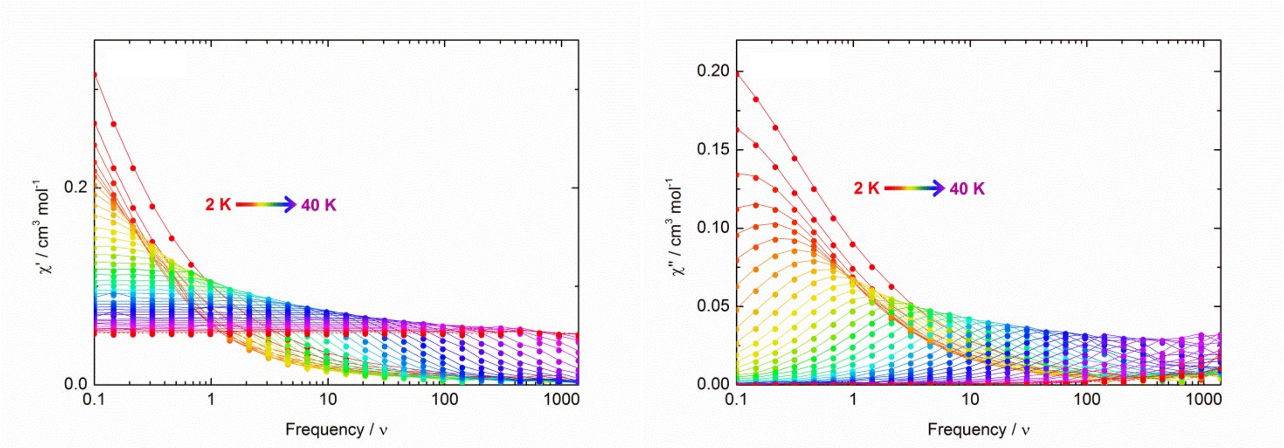

Figure S15. Frequency dependence of the in-phase (χ') and the out-of-phase (χ'') magnetic susceptibility for **Dy@6-Y** in a matrix of **6-Y** (1:20 Dy:Y). Data collected using an oscillating field of H_{ac} = 1.55 Oe and zero applied field.

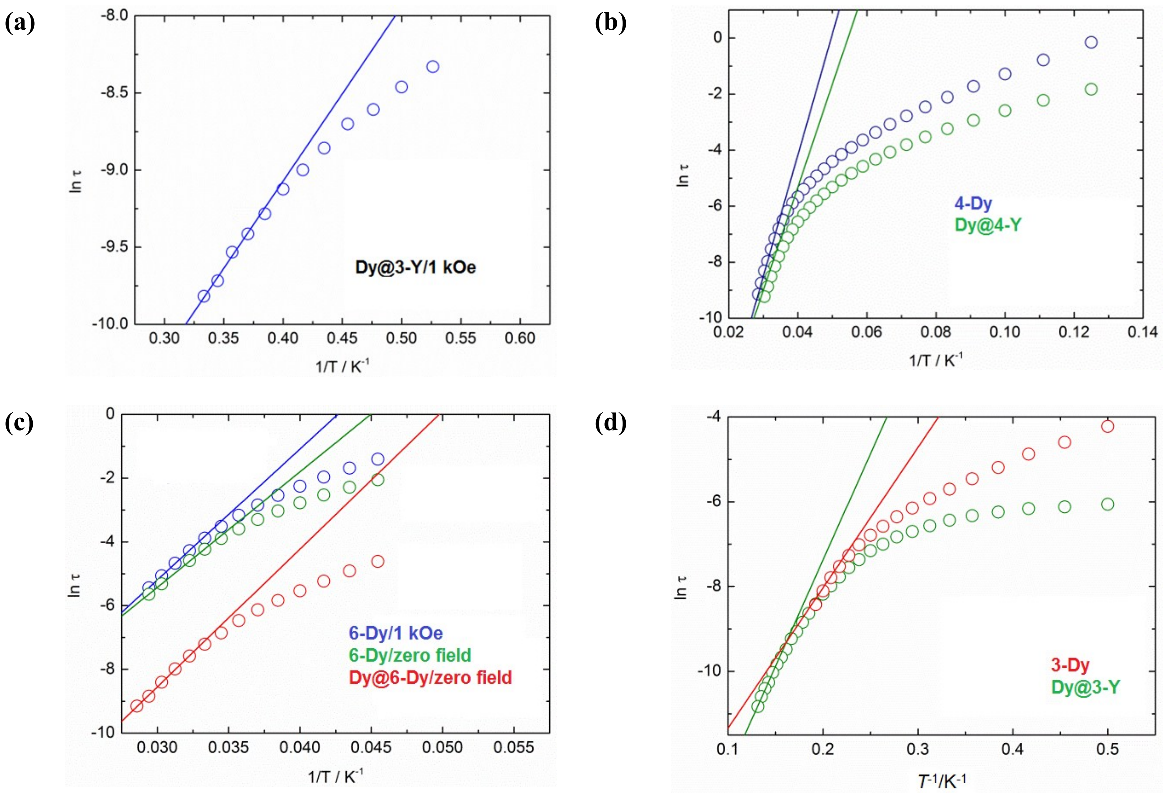

Figure S16. Arrhenius plots of ln τ vs. $1/T$ for: (a) **Dy@3-Y**; (b) **4-Dy**·toluene and **Dy@4-Y**·toluene; (c) **6-Dy**·toluene (H_{dc} = 0 and H_{dc} = 1 kOe) and **Dy@6-Y**·toluene; (d) $[Li(thf)_4]_2$[**3-Dy**]·thf and $[Li(thf)_4]_2$[**Dy@3-Y**]·thf. The solid lines correspond to fits of the high temperature data: U_{eff} and τ_0 values are displayed in the graphs.

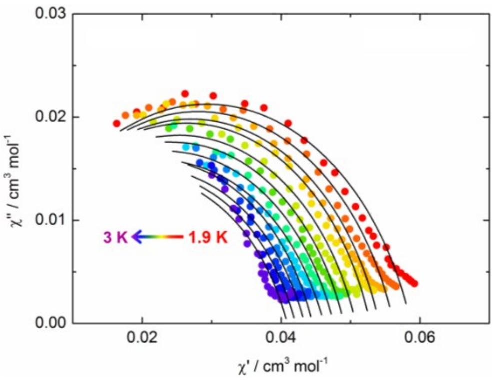

Figure S17. χ' vs. χ'' for **Dy@3-Y** in H_{dc} = 1 kOe. Solid lines are fits to the experimental data.

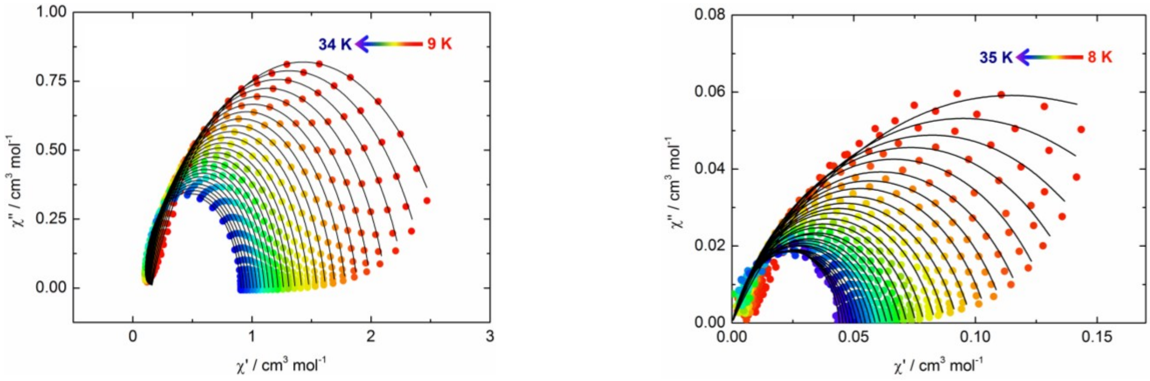

Figure S18. χ' vs. χ'' for **4-Dy** (left) and **Dy@4-Y** (right). Solid lines are fits to the experimental data.

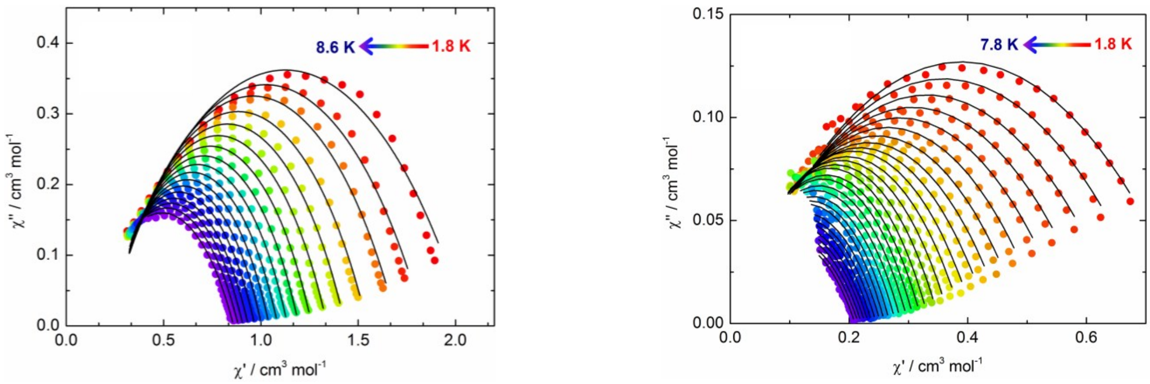

Figure S19. χ' vs. χ'' for **5-Dy** (left) and **Dy@5-Y** (right). Solid lines are fits to the experimental data.

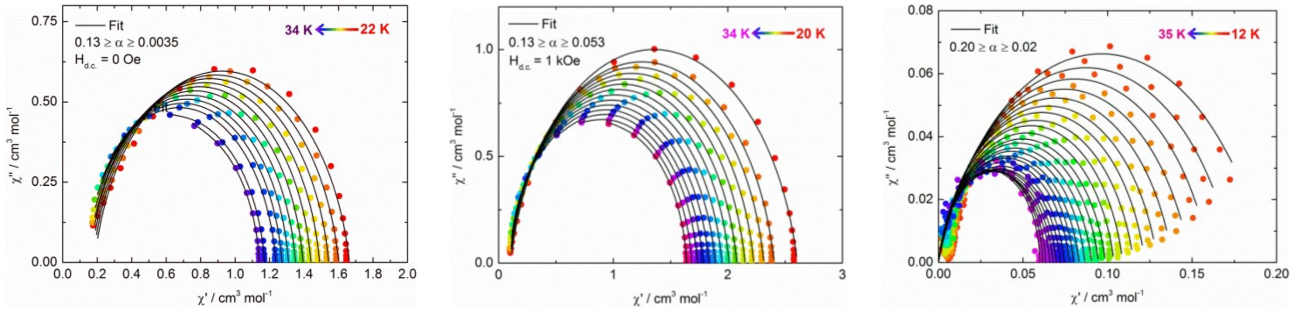

Figure S20. χ' vs. χ'' for **6-Dy** in zero d.c. field (left); for **6-Dy** in H_{dc} = 1 kOe (centre); and **Dy@6-Y** in zero d.c. field (right). Solid lines are fits to the experimental data.

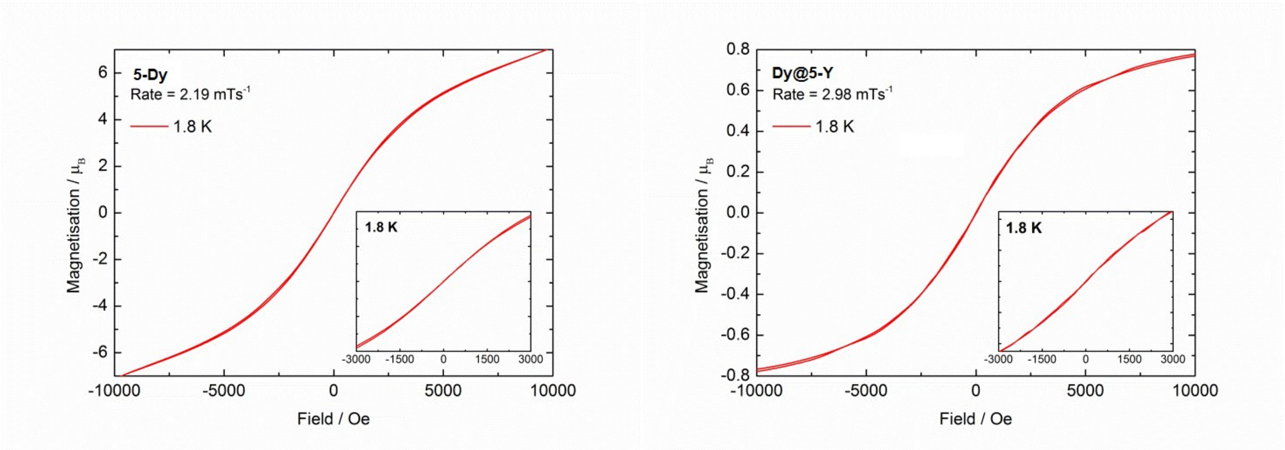

Figure S21. Field (H) dependence of the magnetization (M) for undiluted [Li(thf)$_4$]$_2$[**5-Dy**]·thf and [Li(thf)$_4$]$_2$ [**Dy@3-Y**]·thf with H = ±10000 Oe. Inset: expansion of the regions with H = ±3000 Oe.

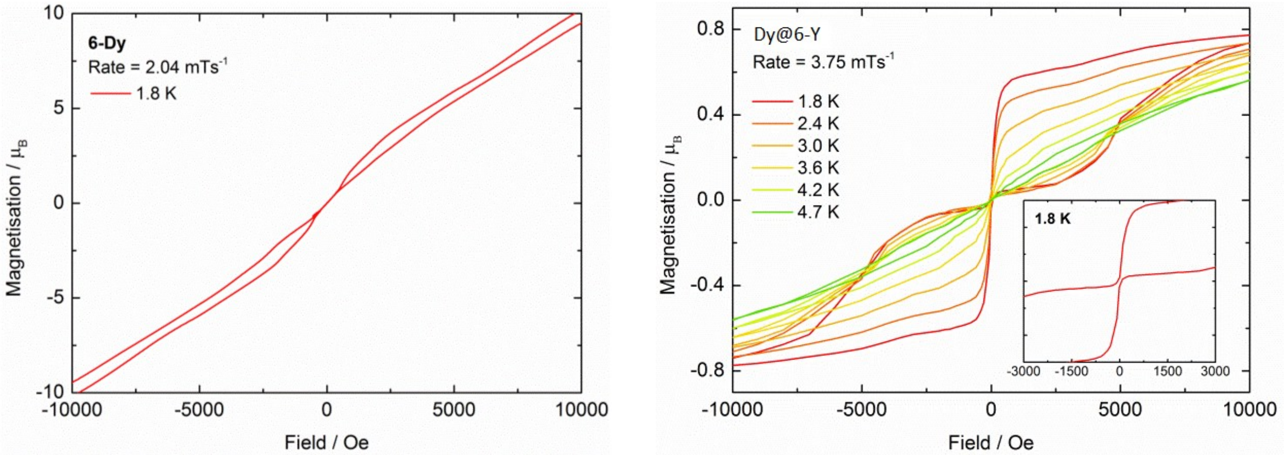

Figure S22. Field (H) dependence of the magnetization (M) for undiluted **6-Dy** (left) and diluted **Dy@6-Y** (right). Inset: expansion of the region with $H = \pm 3000$ Oe.

Computational details

All calculations were carried out with MOLCAS 8.0 and are of CASSCF/RASSI/SINGLE_ANISO type. The Cholesky decomposition threshold was set to 5×10^{-8} to save disk space. Each magnetic center was calculated keeping the experimental observed geometry of the entire molecule and just replacing the Dy(III) ions by diamagnetic Lu(III) ions.

Two basis set approximations have been employed: basis 1 – small, and basis 2 – large. Table 1 shows the contractions of the employed basis sets for all elements.

Table S2. Contractions of the employed basis sets in computational approximations 1 and 2.

Basis 1	Basis 2
Dy.ANO-RCC-VDZP.	Dy.ANO-RCC-VTZP.
Lu.ANO-RCC-VDZP.	Lu.ANO-RCC-VTZP.
As.ANO-RCC-VDZP. (close)	As.ANO-RCC-VTZP. (close)
As.ANO-RCC-MB. (distant)	As.ANO-RCC-VDZP. (distant)
Se.ANO-RCC-VDZP. (close)	Se.ANO-RCC-VTZP. (close)
Se.ANO-RCC-MB. (distant)	Se.ANO-RCC-VDZP. (distant)
Li.ANO-RCC-MB.	Li.ANO-RCC-VDZP.
C.ANO-RCC-VDZP. (close)	C.ANO-RCC-VTZP. (close)
C.ANO-RCC-MB. (distant)	C.ANO-RCC-MB. (distant)
H.ANO-RCC-VDZP. (close)	H.ANO-RCC-VTZP.
H.ANO-RCC-MB. (distant)	H.ANO-RCC-MB.

The active space of the CASSCF method included 9 electrons in 7 orbitals (4*f* orbitals of Dy^{3+} ion).
We have mixed 21 sextets, 128 quartet and 130 doublet states by spin-orbit coupling.
On the basis of the resulting spin-orbital multiplets SINGLE_ANISO program computed local magnetic properties (*g*-tensors, magnetic axes, local magnetic susceptibility, etc.).

Broken-Symmetry DFT calculations were carried out using the ORCA 3.0.0 program. The Dy(III) ions were replaced with Gd(III), while the position of other atoms were kept as in the experimentally determined structure. The exchange coupling parameters were derived by employing the generalized algorithm for calculation of Heisenberg exchange constants in multispin systems.[5] The calculated *J(*Gd-Gd) parameters were rescaled from the spin 7/2 to the Dy spin 5/2 in order to get the *J*(Dy-Dy) parameter. This was done by multiplying the *J(*Gd-Gd) parameter by a factor of 49/25.

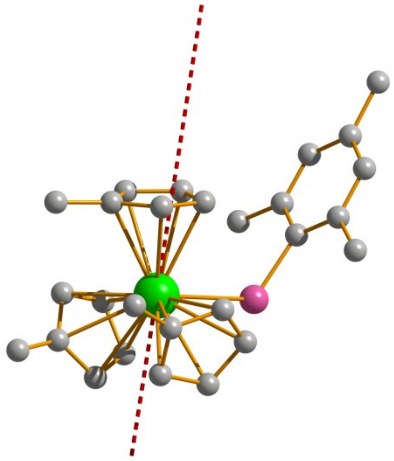

Figure S23. Structure of **3-Dy**. The hydrogen atoms were removed for clarity. The dashed line shows the orientation of the main magnetic axis in the ground Kramers doublet. Green = Dy, purple = As, grey = C.

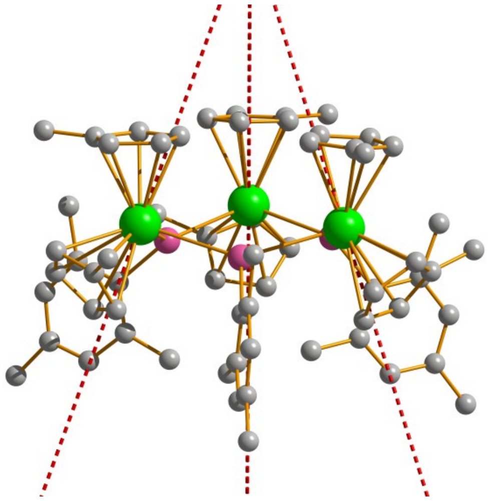

Figure S24. Structure of **4-Dy**. The hydrogen atoms were removed for clarity. The dashed lines show the orientation of the main magnetic axes in the ground Kramers doublets. Green = Dy, purple = As, grey = C.

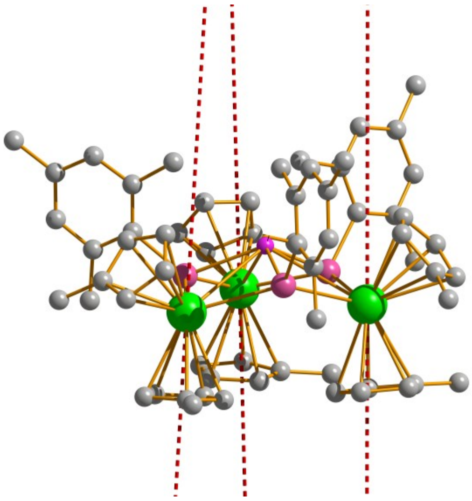

Figure S25. Structure of **5-Dy**. The hydrogen atoms were removed for clarity. The dashed lines show the orientation of the main magnetic axes in the ground Kramers doublets. Green = Dy, purple = As, pink = Li, grey = C.

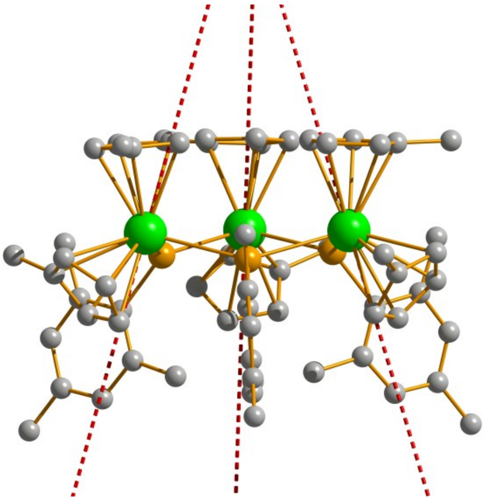

Figure S26. Structure of Dy_3Se_3 compound. The hydrogen atoms were removed for clarity. The dashed lines show the orientation of the main magnetic axes in the ground Kramers doublets. Green = Dy, orange = Se.

Table S3. Energies of the lowest Kramers doublets of the Dy(III) centre in **3-Dy**.

**Spin-orbit energies, cm^{-1}**	
**basis1**	**basis2**
0.000	0.000
43.093	43.865
103.342	105.323
283.102	290.270
345.148	351.304
407.612	411.559
479.230	480.678
624.282	626.429

Table S4. The *g* tensors of the lowest Kramers doublets (KD) of Dy center in **3-Dy**.

**KD**		**basis1** ***g***	**basis2** ***g***
**1**	g_X	1.007638	1.031472
	g_Y	6.451373	6.895653
	g_Z	14.342077	13.921937
**2**	g_X	0.786326	0.644982
	g_Y	2.891924	2.933299
	g_Z	7.107531	6.361214
**3**	g_X	2.760593	2.756997
	g_Y	5.240434	5.539521
	g_Z	11.318941	11.039960
**4**	g_X	5.464831	5.468013
	g_Y	6.986599	7.215991
	g_Z	9.480995	9.295032

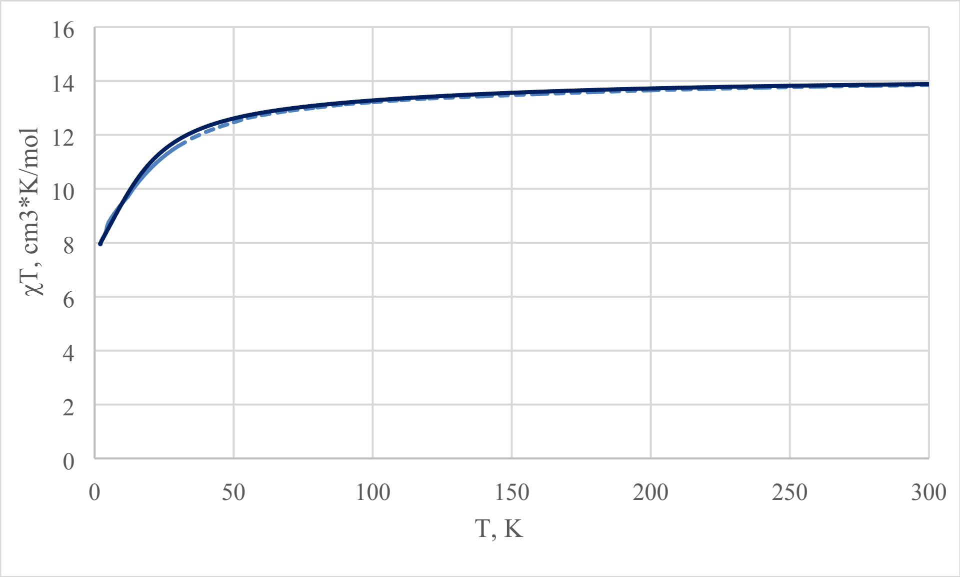

Figure S27. Experimental (dashed line, up-scaled by 4%) and calculated $\chi_M T$ vs T for **3-Dy**.

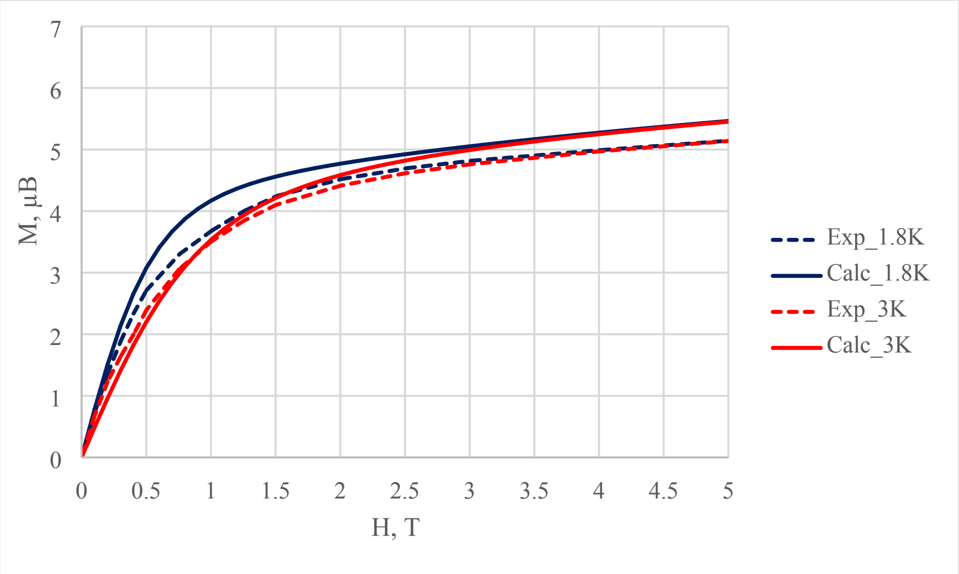

Figure S28. Experimental and calculated magnetization vs. field for **3-Dy** at 1.8 and 3 K.

Table S5. Energies of the lowest Kramers doublets (cm^{-1}) of Dy centers in **4-Dy**.

Spin-orbit energies, cm^{-1}					
**Dy1_basis1**	**Dy1_basis2**	**Dy2_basis1**	**Dy2_basis2**	**Dy3_basis1**	**Dy3_basis2**
0.000	0.000	0.000	0.000	0.000	0.000
134.427	143.918	132.937	142.303	135.839	146.128
282.245	299.682	292.603	310.597	280.784	300.260
363.809	384.472	379.051	400.506	363.525	386.727
380.043	402.242	390.606	412.587	416.348	441.551
419.249	441.500	437.265	460.439	456.214	481.739
451.982	476.196	478.244	504.243	485.766	510.312
575.642	609.399	612.920	649.835	558.399	593.714

Table S6. The *g* tensors of the lowest Kramers doublets (KD) of Dy centers in **4-Dy**.

KD		Dy1_basis1	Dy1_basis2	Dy2_basis1	Dy2_basis2	Dy3_basis1	Dy3_basis2
		g	*g*	*g*	*g*	*g*	*g*
**1**	g_X	0.00003	0.000064	0.000066	0.000118	0.000270	0.000333
	g_Y	0.00005	0.000093	0.000127	0.000169	0.000365	0.000449
	g_Z	19.53414	19.536717	19.546143	19.551494	19.599571	19.602727
**2**	g_X	0.000240	0.000391	0.000470	0.000421	0.002429	0.003177
	g_Y	0.000294	0.000467	0.000588	0.000575	0.002902	0.003772
	g_Z	17.090793	17.080292	17.050366	17.038135	17.147046	17.133962
**3**	g_X	0.011795	0.021103	0.029854	0.031334	0.042254	0.052732
	g_Y	0.012279	0.021196	0.032806	0.035291	0.061877	0.075685
	g_Z	14.939508	14.929432	14.840380	14.824766	14.806272	14.804128
**4**	g_X	0.940020	0.952060	1.347636	1.218157	1.338162	1.486674
	g_Y	4.549598	4.582977	4.382033	4.404079	1.894322	2.074917
	g_Z	9.459901	9.343986	9.949418	10.227908	10.977326	10.934034

Table S7. Energies of the lowest Kramers doublets (cm^{-1}) of Dy centers in **5-Dy**.

Spin-orbit energies, cm^{-1}					
**Dy1_basis1**	**Dy1_basis2**	**Dy2_basis1**	**Dy2_basis2**	**Dy3_basis1**	**Dy3_basis2**
0.000	0.000	0.000	0.000	0.000	0.000
103.150	102.475	68.357	72.460	75.716	75.649
124.561	135.258	102.148	122.135	93.719	102.178
138.109	150.957	121.411	137.313	129.893	147.853
164.220	177.870	151.791	170.029	140.773	155.407
179.967	194.110	169.313	183.607	155.591	170.987
212.177	240.372	184.245	211.048	194.859	221.772
298.471	305.308	269.537	282.045	234.141	247.391

Table S8. The *g* tensors of the lowest Kramers doublets (KD) of Dy centers in **5-Dy**.

KD		Dy1_basis1	Dy1_basis2	Dy2_basis1	Dy2_basis2	Dy3_basis1	Dy3_basis2
		g	*g*	*g*	*g*	*g*	*g*
1	g_X	0.006516	0.003313	0.012125	0.003849	0.003045	0.002724
	g_Y	0.008034	0.004483	0.022233	0.008082	0.004109	0.003530
	g_Z	19.623364	19.618450	19.256045	19.253434	19.413266	19.345959
2	g_X	0.086535	0.044776	0.064433	0.037739	0.114768	0.123225
	g_Y	0.133842	0.059965	0.146189	0.075556	0.200885	0.182161
	g_Z	18.810018	18.820514	18.414234	18.217508	18.361544	18.142061
3	g_X	1.314095	0.691297	0.071469	0.294926	0.121084	0.101548
	g_Y	5.533295	3.507187	0.517180	1.923003	0.649171	0.445357
	g_Z	12.575944	15.057778	15.466483	15.421349	17.531795	17.888337
4	g_X	0.333906	0.436704	2.007959	2.238581	4.679485	7.913623
	g_Y	4.925088	1.914114	5.071901	5.008151	4.851304	5.957034
	g_Z	9.573488	12.508223	13.186224	11.949750	8.949160	0.977835

Table S9. Energies of the lowest Kramers doublets (cm^{-1}) of Dy centers in **6-Dy**.

Spin-orbit energies, cm^{-1}					
**Dy1_basis1**	**Dy1_basis2**	**Dy2_basis1**	**Dy2_basis2**	**Dy3_basis1**	**Dy3_basis2**
0.000	0.000	0.000	0.000	0.000	0.000
144.342	150.307	134.184	140.401	145.371	152.664
299.988	313.467	286.411	300.633	297.523	311.472
389.166	404.280	371.715	387.803	384.821	400.700
432.514	448.690	414.372	428.765	440.942	458.592
462.643	478.948	440.865	457.685	460.752	480.552
504.048	518.066	481.941	495.850	496.589	514.128
581.281	603.296	551.279	576.356	569.372	591.990

Table S10. The *g* tensors of the lowest Kramers doublets (KD) of Dy centers in **6-Dy**.

KD		Dy1_basis1	Dy1_basis2	Dy2_basis1	Dy2_basis2	Dy3_basis1	Dy3_basis2
		g	*g*	*g*	*g*	*g*	*g*
1	g_X	0.000104	0.000112	0.000132	0.000189	0.000030	0.000001
	g_Y	0.000137	0.000148	0.000160	0.000223	0.000040	0.000016
	g_Z	19.618673	19.603185	19.508777	19.485266	19.614191	19.607513
2	g_X	0.000880	0.000887	0.000636	0.000881	0.000445	0.000239
	g_Y	0.001059	0.001093	0.000813	0.001130	0.000579	0.000355
	g_Z	17.189153	17.161314	17.154409	17.120763	17.153968	17.144021
3	g_X	0.008409	0.010258	0.020693	0.028361	0.008231	0.011710
	g_Y	0.011490	0.013270	0.030680	0.040005	0.010556	0.014143
	g_Z	14.779376	14.785407	14.751254	14.761951	14.782179	14.793340
4	g_X	0.442573	0.628316	0.413941	0.645820	0.597336	0.798065
	g_Y	0.700690	0.972565	0.582530	0.934593	0.701903	0.903728
	g_Z	11.474231	11.332165	11.397447	11.241813	11.436819	11.341716

Total magnetic interactions in 4-Dy, 5-Dy and 6-Dy

The following exchange Hamiltonian was used to account for total magnetic interaction:

$$\hat{H} = -[(J_{12}^{dip} + J_{12}^{exch})\hat{\tilde{S}}_{1,z1}\hat{\tilde{S}}_{2,z2} + (J_{13}^{dip} + J_{13}^{exch})\hat{\tilde{S}}_{1,z1}\hat{\tilde{S}}_{3,z3} + (J_{23}^{dip} + J_{23}^{exch})\hat{\tilde{S}}_{2,z2}\hat{\tilde{S}}_{3,z3}]$$

The Ising exchange parameters were calculated from Lines parameters by the expression:

$$J_{ij}^{Ising} = 25 J_{Lines} \cos\varphi_{ij}$$

where φ_{ij} is the angle between the main anisotropy axes of the interacting sites.

The Lines parameters have been determined by fitting the experimental magnetic susceptibility data (Figures 7, 9, 11). The dipolar parameters were calculated straightforwardly.

Table S11. Fitted Lines parameters *vs.* DFT parameters (cm^{-1}).

Complex	Pair	Lines parameter	DFT
**4-Dy**	Dy1-Dy2	-0.2	-0.7
	Dy1-Dy3	-0.3	-0.6
	Dy2-Dy3	-0.2	-0.5
**5-Dy**	Dy1-Dy2	-0.2	-0.3
	Dy1-Dy3	-0.3	-0.3
	Dy2-Dy3	-0.4	-0.4
**6-Dy**	Dy1-Dy2	-0.2	-0.3
	Dy1-Dy3	-0.2	-0.3
	Dy2-Dy3	-0.2	-0.3

Table S12. Exchange interactions between Dy ions in **4-Dy**, Ising parameters (cm^{-1}):

Molecule/approximation		J_{dip}*	J_{exch}
**basis1**	Dy1-Dy2	-1.08	-3.99
	Dy1-Dy3	-1.15	-5.72
	Dy2-Dy3	-1.08	-3.84

* contribution arising only from the Ising terms $\sim \hat{\tilde{S}}_{1,z}\hat{\tilde{S}}_{2,z}$ to the dipolar coupling. In the calculation of the exchange spectrum the dipolar interaction included all terms.

Table S13. Exchange interactions between Dy ions in **5-Dy,** Ising parameters (cm^{-1}):

Molecule/approximation		J_{dip}*	J_{exch}
**basis1**	Dy1-Dy2	-1.12	-5.49
	Dy1-Dy3	-1.11	-6.67
	Dy2-Dy3	-1.09	-8.67

Table S14. Exchange interactions between Dy ions in **6-Dy,** Ising parameters (cm^{-1}):

Molecule/approximation		J_{dip}*	J_{exch}
**basis1**	Dy1-Dy2	-1.15	-3.61
	Dy1-Dy3	-1.21	-3.57
	Dy2-Dy3	-1.17	-3.85

Table 14. Energies (cm^{-1}) of the lowest four exchange doublet states considering the total magnetic interaction.

Basis 2		
**4-Dy**	**5-Dy**	**6-Dy**
0.0000	0.0000	0.0000
0.0622	0.5577	0.0117
0.9069	1.3905	0.1216
5.6244	8.0905	4.6280

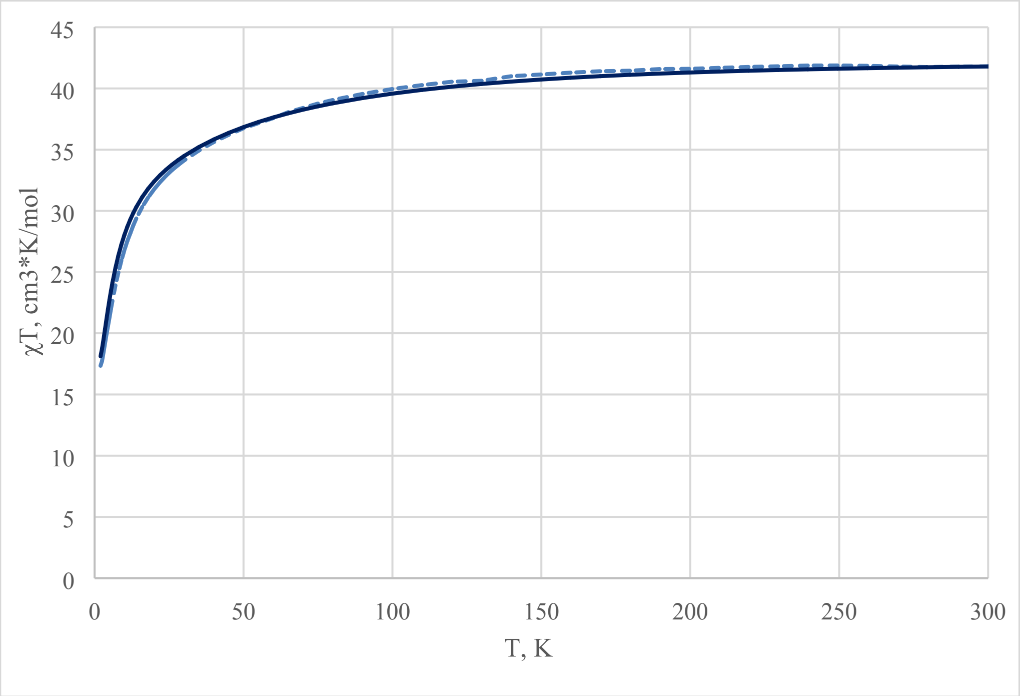

Figure S29. Experimental (dashed line, down-scaled by 4%) and calculated $\chi_M T$ vs T for **4-Dy**.

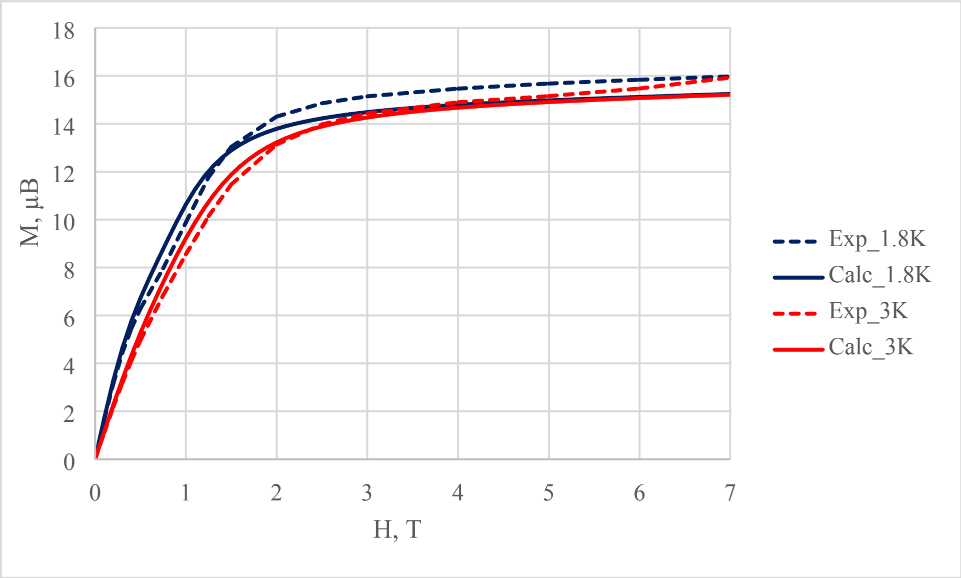

Figure S30. Experimental and calculated magnetization vs. field for **4-Dy** at 1.8 and 3 K.

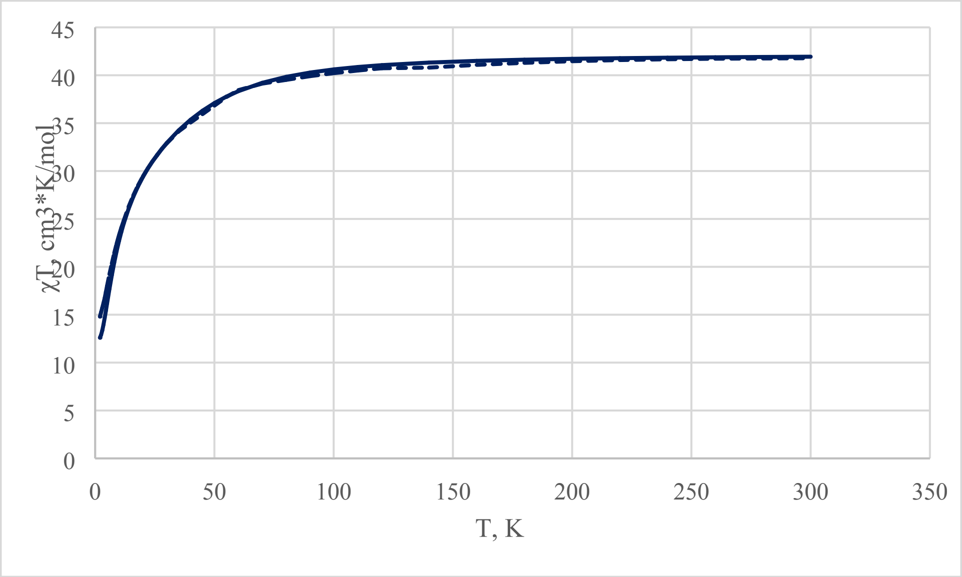

Figure S31. Experimental (dashed line) and calculated $\chi_M T$ vs T for **5-Dy**.

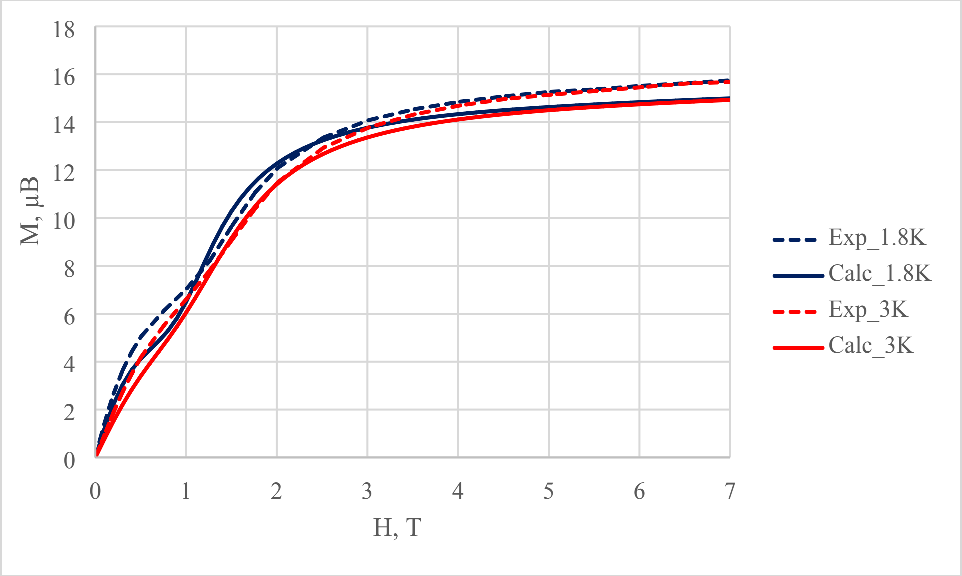

Figure S32. Experimental and calculated magnetization vs. field for **5-Dy** at 1.8 and 3 K.

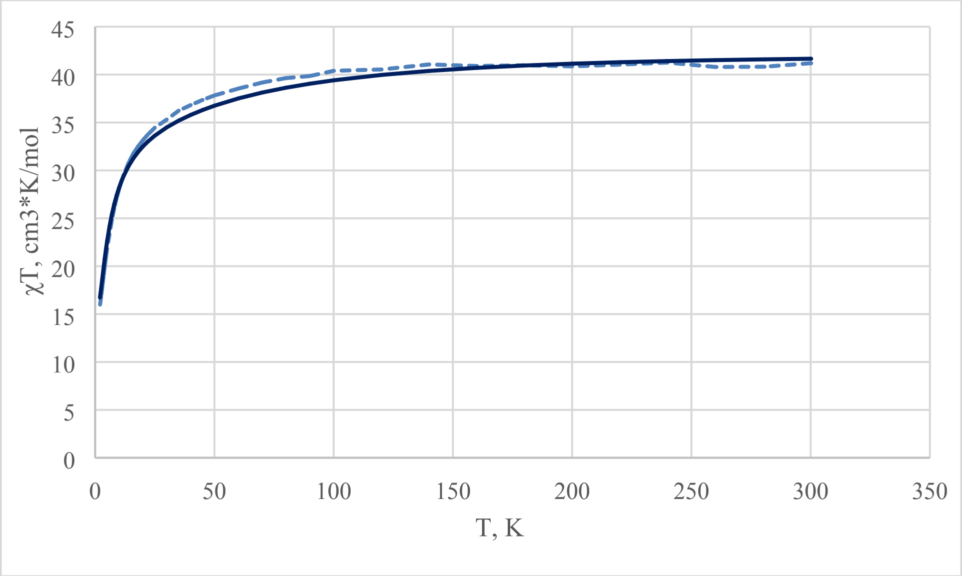

Figure S33. Experimental (dashed line, down-scaled by 4%) and calculated $\chi_M T$ vs T for **6-Dy**.

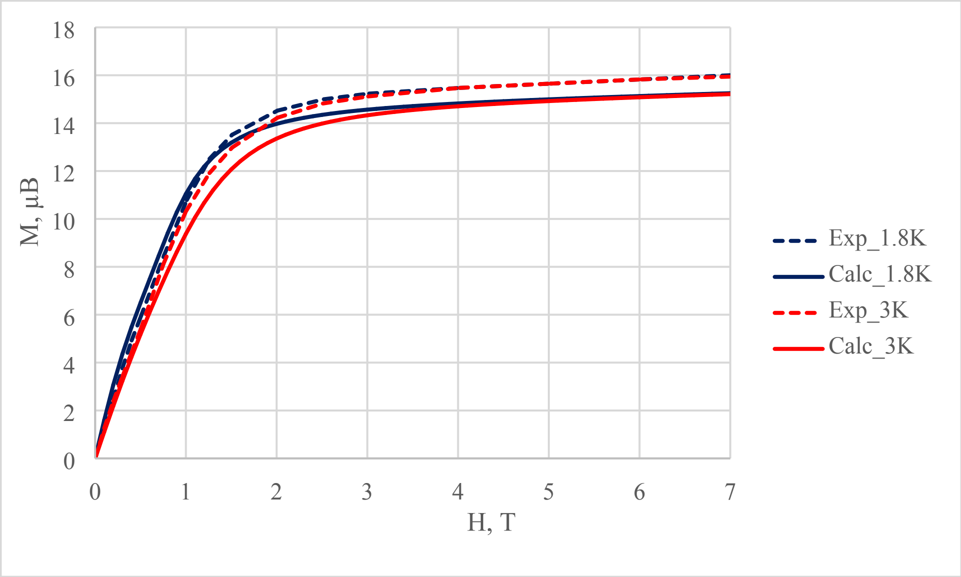

Figure S34. Experimental and calculated magnetization vs. field for **6-Dy** at 1.8 and 3 K.

References

1) T. Pugh, A. Kerridge and R. A. Layfield, *Angew. Chem. Int. Ed.*, **2015**, *54*, 4255.
2) G. Wilkinson and J. M. Birmingham, *J. Am. Chem. Soc.*, **1954**, *76*, 6210.
3) R. V. Bonnert and P. R. Jenkins, *J. Chem. Soc., Perkin Trans. 1,* **1989**, 413.
4) C. Wombwell and E. Reisner, *Dalton Trans*, **2014**, *43*, 4483.
5) M. Shoji, K. Koizumi, Y. Kitagawa, T. Kawakami, S. Yamanaka, M. Okumura, K. Yamaguchi, *Chem. Phys. Lett.* **2006,** 432, 343.
